# Generation of Isogenic hiPSCs with Targeted Edits at Multiple Intronic SNPs to Study the Effects of the Type 2 Diabetes Associated *KCNQ1* Locus in American Indians

**DOI:** 10.3390/cells11091446

**Published:** 2022-04-25

**Authors:** Anup K. Nair, Michael Traurig, Jeff R. Sutherland, Yunhua L. Muller, Emma D. Grellinger, Lucas Saporito, Robert G. Nelson, Clifton Bogardus, Leslie J. Baier

**Affiliations:** Phoenix Epidemiology and Clinical Research Branch, National Institute of Diabetes and Digestive and Kidney Diseases, National Institutes of Health, 445 N 5th Street, Phoenix, AZ 85004, USA; anup.nair@nih.gov (A.K.N.); mtraurig@phx.niddk.nih.gov (M.T.); jsutherlan@phx.niddk.nih.gov (J.R.S.); ymuller@mail.nih.gov (Y.L.M.); emma.grellinger@nih.gov (E.D.G.); lsaporito10@gmail.com (L.S.); rnelson@phx.niddk.nih.gov (R.G.N.); cbogardus@phx.niddk.nih.gov (C.B.)

**Keywords:** *KCNQ1*, type 2 diabetes, hiPSCs, pancreatic-beta like cells, *CDKN1C*, *H19*, allele-specific guide RNA

## Abstract

The top genetic association signal for type 2 diabetes (T2D) in Southwestern American Indians maps to intron 15 of *KCNQ1*, an imprinted gene. We aim to understand the biology whereby variation at this locus affects T2D specifically in this genomic background. To do so, we obtained human induced pluripotent stem cells (hiPSC) derived from American Indians. Using these iPSCs, we show that imprinting of *KCNQ1* and *CDKN1C* during pancreatic islet-like cell generation from iPSCs is consistent with known imprinting patterns in fetal pancreas and adult islets and therefore is an ideal model system to study this locus. In this report, we detail the use of allele-specific guide RNAs and CRISPR to generate isogenic hiPSCs that differ only at multiple T2D associated intronic SNPs at this locus which can be used to elucidate their functional effects. Characterization of these isogenic hiPSCs identified a few aberrant cell lines; namely cell lines with large hemizygous deletions in the putative functional region of *KCNQ1* and cell lines hypomethylated at the *KCNQ1OT1* promoter. Comparison of an isogenic cell line with a hemizygous deletion to the parental cell line identified *CDKN1C* and *H19* as differentially expressed during the endocrine progenitor stage of pancreatic-islet development.

## 1. Introduction

Genome-wide association studies (GWAS) and subsequent meta-analyses have identified hundreds of loci that associate with type 2 diabetes (T2D) in various populations [1,2,3,4,5]. Most of the identified loci appear to impact T2D via an effect on insulin secretion and many associated SNPs map to enhancers that are active in pancreatic islets [6,7,8,9]. In American Indians from the southwestern region of the United States, the strongest T2D signal identified to date maps to intron 15 of *KCNQ1* [10]. Among normal glucose tolerant American Indians, variants that increase risk for T2D show a strong association with insulin secretory responses to either oral glucose or an intravenous glucose bolus indicating that *KCNQ1* variants impact T2D risk via an effect on insulin secretion [10]. These associations have a strong parent-of-origin effect, whereby only maternal inheritance of the risk allele associated with increased risk for T2D and a lower insulin secretion in response to a glucose challenge [10]. Variation at the *KCNQ1* locus has also been shown to associate with T2D in diverse ethnic groups [10,11,12,13,14,15] with studies proposing that the diabetes risk effects are mediated during early development [16]. However, the effector gene mediating this phenotype and the pathomechanism by which these SNPs affect T2D risk remain elusive, in part due to a lack of a biological model system.

The *KCNQ1* locus is located in an imprinted, gene rich region. Many genes in this region, including *CDKN1C, TRPM5, IGF2, TH, H19,* and *INS,* are known to be important for pancreatic-islet development and function. Imprinting in this region is controlled by two distinct imprinting control regions (ICR). One ICR is immediately 5′ to H19 and the other ICR is in intron 10 of *KCNQ1* which controls the imprinting of genes including *KCNQ1, KCNQ1OT1, CDKN1C* and *TRPM5* [17]. A previous study in adult islet and fetal pancreas found evidence of imprinting for *KCNQ1* and *KCNQ1OT1* only in fetal pancreas. In contrast *CDKN1C* was imprinted in both adult islet and fetal pancreas, whereas *PHLDA2*, *SLC22A18* and *SLC22A18AS* were biallelically expressed in both fetal and adult pancreas. This led to the suggestion that *KCNQ1*, *KCNQ1OT1* or *CDKN1C* is the probable effector gene at this locus. This study also identified correlation between a representative T2D SNP (rs2237895) at this locus and methylation at three sites (CTCF and PLAGL1 binding sites and a diagnostic differentially methylated region used for diagnosis of Beckwith-Wiedemann syndrome) at different developmental stages leading the authors to suggest that the diabetes risk effects may be mediated during early development [16].

Recent advances in the generation of pancreatic beta-like cells from human induced pluripotent stem cells (hiPSCs) provide an ideal model system to study the developmental stage-specific effect of T2D associated variants at the *KCNQ1* locus [18,19,20,21,22,23]. Pancreatic beta-like cells generated from genome edited, isogenic hiPSCs differing only at the SNPs of interest will advance our understanding of the biologic impact of these variants. However, a challenge for these studies is the high linkage disequilibrium (LD) between the lead variant at this locus and several nearby variants, which may be the true causal variant for the observed functional effect. This uncertainty of the causal variant at this locus necessitates multiple edits in the same cell line to generate isogenic hiPSCs. In addition, the *KCNQ1* locus has a particularly strong association with T2D in Southwestern American Indians [10,24]; therefore, to better understand the physiologic processes that connect variation at this locus to risk of T2D in this specific ethnic group, SNPs should be studied in the context of the genomic background of Southwestern American Indians. In this report, we describe the use of allele-specific guide RNAs to target the *KCNQ1* region and establishment of isogenic cell lines generated using hiPSCs procured from Southwestern American Indians that differ only at multiple T2D associated *KCNQ1* intronic SNPs. As a proof of concept, we also provide evidence that the targeted region has developmental stage specific regulatory function by differentiating an isogenic hiPS cell line with a hemizygous deletion towards pancreatic beta-like cells. We plan to use our established isogenic hiPSCs with targeted edits at the T2D associated SNPs in future studies to examine the pathomechanism by which these SNPs affect T2D risk in American Indians.

## 2. Materials and Methods

### 2.1. Induced Pluripotent Stem Cell Derivation and Culture

hiPSCs were derived as a part of a project to study diabetic kidney diseases in American Indians. These subjects were previously clinically studied, and their DNA had undergone whole-exome sequencing and genome-wide SNP genotyping. hiPSCs used in this study were derived from female subjects who were obese (BMI > 30 kg/m^2^) and were diagnosed with type 2 diabetes. hiPSCs were derived from peripheral blood mononuclear cells, collected at the clinical center, which were then reprogrammed into hiPSCs at the NIH-NHLBI iPSC core facility using a Sendai virus based reprogramming kit (Thermo Fisher, Waltham, MA, USA). The hiPSCs were initially expanded using mTeSR1 medium (cat. No. 85850, Stem Cell Technologies, Vancouver, BC, Canada) and Matrigel (cat. No. 354227, Corning, Corning, NY, USA). Sendai virus free and mycoplasma negative hiPSCs were then adapted (5 passages) and expanded using DEF-CS 500 culture system (cat No. Y30010, Takara Bio, San Jose, CA, USA), a chemically defined, feeder free system for the expansion of hiPSCs in a 2D-monlayer format. Upon reaching dense confluency, the cells were passaged every three days using TrypLE select (cat. No. 12563011, Thermo Fisher). These hiPSCs were further characterized for pluripotency, differentiation potential and genetic stability as detailed below (Figure S1).

### 2.2. Differentiating hiPSCs to Beta-like Cells

Detailed methods used for differentiation of hiPSCs to beta-like cells is provided below. Briefly, two different protocols were used for generating beta-like cells from hiPSCs. Protocol 1 utilized a slightly modified hybrid adherent/air-liquid interphase culture system described by Rezania et al. [18]. Protocol 1 was used in our early studies which measured expression levels for *KCNQ1* and neighboring genes and assessed allele-specific expression during beta-like cell differentiation from hiPSCs. Although protocol 1 generated beta-like cells and resulted in proper expression of markers during different stages of differentiation, this protocol resulted in highly variable differentiation efficiency (Figure S2) and generated lower numbers of beta-like cells from our American Indian hiPSCs. Therefore, a second protocol (Protocol 2) was optimized for our hiPSCs and was used for all other differentiations. Protocol 2 followed a hybrid adherent/3D culture system (Figure S3A) and was developed based on previously published protocols [20,21,22,23].

The main features of protocol 2 are (1) the use of iMatrix-511 (highly purified and refined laminin-511 E8 fragments, cat. No. NP892-012, Stemgent, Cambridge, MA, USA) as the basement membrane matrix, (2) the use of a WNT signaling inhibitor (cat. No. 72122, IWP2, Stem Cell Technologies) and a shorter duration (48 h vs. 72 h) for the generation of definitive endoderm, (3) the use of Epidermal Growth Factor (EGF, cat. No. 236-EG-200, R & D Systems, Minneapolis, MN, USA) for the generation of pancreatic progenitors, and (4) the use of N-acetyl Cysteine (cat. No. A9165-25G, Sigma, St. Louis, MO, USA) during the early endocrine progenitor stage. This optimized differentiation protocol consistently generated high quality definitive endoderm cells after two days of differentiation and >80% PDX1/NKX6-1 double positive pancreatic progenitor cells after 10 days of differentiation as an adherent monolayer (Figure S3B,C). Further differentiation as 3D clusters generated monohormonal insulin (beta-like cells) positive cells co-expressing beta-cell transcription factors such as NKX6-1 and PDX1 (Figure S3D–F). This modified protocol leads to consistent generation of >50% monohormonal INS/NKX6-1 double positive beta-like cells (functional beta-like cells) after 34 days of differentiation (Figure S3G,H) that respond to a high glucose challenge by increasing insulin secretion during a static glucose-stimulated insulin secretion assay (Figure S3I).

Protocol 1: Modified from Rezania et al. [18].

hiPSCs maintained in DEF-CS 500 culture system were seeded in iMatrix-511 (Stemgent) coated plates at a density of 1 × 10^5^ cells/cm^2^. Differentiation was initiated after 48 h. using Growth Differentiation Factor-8 (GDF8, cat. No. 120-00, Peprotech, Cranbury, NJ, USA) and the WNT signaling activator CHIR99021 (cat. No. S2924, Selleckchem, Houston, TX, USA) to generate definitive endoderm cells (day 3). These cells were then differentiated towards pancreatic progenitors (day 10) using a combination of ascorbic acid (cat. No. A4544, Sigma), FGF7 (cat. No. 251-KG-01M, R & D Systems), LDN193189 (cat. No. 04-0074-02, Stemgent), SANT-1 (cat. No. S4572, Sigma), TPPB (cat. No. 5343, Tocris, Bristol, UK), retinoic acid (cat. No. R2625, RA, Sigma) and EGF (R and D systems). On day 10, cells were harvested using TrypLE select (Thermo Fisher) and resuspended at a density of 5 × 10^7^ cells/mL. Five microliters of cells were then spotted on a Falcon cell culture insert (cat. No. 353102, 1.0 µm pore size, Corning) and were further differentiated at an air/liquid interphase towards immature beta-like cells (Day 20) using a combination of γ-Secretase Inhibitor XX (cat. No. 565789, GSiXX, MilliporeSigma, Burlington, MA, USA), ALK5 inhibitor II (cat. No. ALX-270-445-M005, Enzo life sciences, Farmingdale, NY, USA) and 3,3′,5-Triiodo-L-thyronine (cat. No. T6397, T3, Sigma). Finally, mature beta-like cells (Day 35) were generated using ALK5 inhibitor II, 3,3′,5-Triiodo-L-thyronine, N-acetyl cysteine (Sigma), Trolox (cat. No. 648471, Sigma) and R428 (cat. No. S2841, Selleckchem).

Protocol 2: Optimized seven-stage differentiation protocol.

hiPSCs maintained in DEF-CS 500 culture system were seeded at a density of 7.0 × 10^4^ cells/cm^2^ into iMatrix coated T-75 flasks (cat. No. 0030711114, Eppendorf, Hamburg, Germany). Differentiation was initiated after 72 h. (Day 0). Cells were rinsed with dPBS (on day 0 and day 2) and DEF-CS medium was replaced with the following media on the respective days. Media was changed every day unless otherwise noted. **Day 0**—Basal Differentiation Media 1 (BDM1) + 100 ng/mL Activin A (cat. No. 338-AC-050, R & D Systems) + 3 μM CHIR99021 (Selleckchem) + 100 nM PI103 (cat. No. S1038, Selleckchem). **Day 1**—BDM1 + 100 ng/mL Activin A + 2.5 μM IWP2 (Stem Cell Technologies). **Day 2 and 3**—BDM1 + 0.25 mM ascorbic acid (Sigma) + 50 ng/mL FGF7 (R & D Systems). **Day 4**—BDM2 + 50 ng/mL FGF7 + 100 nM LDN193189 (Stemgent). After 4 h.—BDM2 + 50 ng/mL FGF7 + 100 nM LDN193189 + 0.25 μM SANT-1 (Sigma) + 250 nM TPPB (Tocris) + 2.0 μM RA (Sigma). **Day 5**—BDM2 + 50 ng/mL FGF7 + 0.25 μM SANT-1 + 250 nM TPPB + 2.0 μM RA. **Day 6–9**—BDM2.1 + 2 ng/mL FGF7 + 0.25 μM SANT-1 + 125 μM TPPB+ 0.1 μM RA + 50 ng/mL EGF (R & D Systems). **Day 10**—BDM2.1 + 2 ng/mL FGF7 + 0.25 μM SANT-1 + 125 nM TPPB + 0.1 μM RA + 50 ng/mL EGF + 10 μM Y-27632 (cat. No. 72304, Stem cell technologies). After 4 h. the cells were harvested using TrypLE select (1.5 mL/T75 flask for 15 min at 37 °C) and were resuspended in BDM3.1 containing 0.1 μM RA + 20 ng/mL betacellulin (cat. No. 261-CE-050, R & D Systems) + 1 μM T3 (Sigma) + 0.25 μM SANT1 + 1 μM GSiXX (MilliporeSigma) + 10 μM ALK5 Inhibitor II (Enzo Life Sciences) + 100 nM LDN193189 + 10 μM Y-27632 + 1.0 μM Latrunculin A (cat. No. 10010630, Cayman chemicals, Ann Arbor, MI, USA) at a density of 4 × 10^6^ cells/mL. 5 mL of cells were then added to ultra-low attachment 6-well plates (cat. No. CLS3471-24EA, Corning) and incubated at 37 °C and 5% CO_2_ rotating on a shaker at 100 rpm. On day 11 the aggregates were collected using a 70 micron reversible cell strainer (cat. No. 27260, Stem Cell Technologies) and were transferred to a new ultra-low attachment 6-well plate. Thereafter, media was changed every day by removing 4.5 mL and adding 4.5 mL of new media. **Day 11 and 12**—BDM3 + 0.1 μM RA + 20 ng/mL betacellulin + 1 μM T3 + 0.25 μM SANT1 + 1 μM GSiXX + 10 μM ALK5 inhibitor II + 100 nM LDN193189 + 10 μM Y-27632. **Day 13–17**—BDM3.1 + 25 nM RA + 20 ng/mL betacellulin + 1 μM T3 + 1 μM GSiXX + 10 μM ALK5 inhibitor II + 1 mM N-acetyl cysteine. **Day 18**—Immature islet clusters were collected in 15 mL centrifuge tubes and were washed once with dPBS. Clusters were then incubated in TrypLE Select for 15–20 min at 37 °C. After incubation the cells were gently triturated using a wide bore 1 mL pipette to obtain a single cell suspension. TrypLE was removed by washing with BDM4 and the cells were resuspended in BDM4 containing 1 μM T3 and 1 mM N-acetyl cysteine (islet maturation media) at a concentration of 1.25 × 10^6^ cells/mL. 4 mL of cells were then added to ultra-low attachment 6-well plates and incubated at 37 °C and 5% CO_2_ rotating on a shaker at 100 rpm. **Day 19**—Immature islet clusters were collected using a 70 micron reversible cell strainer and added to a new ultra-low attachment 6-well plate containing 3.5 mL maturation media. Thereafter, media was changed every day by removing 3 mL and adding 3 mL of fresh maturation media until day 27. 

Basal Differentiation Media composition.

Basal Differentiation Media 1 (**BDM1**)—MCDB131 (cat. No. 10372019, Thermo Fisher) + 1.5 g/L NaHCO_3_ (cat. No. S8761, Sigma) + 1X Glutamax (cat. No. 35050061, Thermo Fisher) + 10 mM final glucose (cat No. G8769, Sigma) + 0.5% fatty acid free BSA (cat. No. G8769, Proliant Biologicals, Ankeny, IA, USA).

Basal Differentiation Media 2 (**BDM2**)—MCDB131 + 2.5 g/L NaHCO_3_ + 1X Glutamax + 10 mM final glucose + 2% fatty acid free BSA + 1: 200 ITS-X (cat. No. 51500056, Thermo Fisher) + 0.25 mM ascorbic acid.

Basal Differentiation Media 2.1 (**BDM2.1**)—MCDB131 + 2.5 g/L NaHCO_3_ + 1X Glutamax + 10 mM final glucose + 2% fatty acid free BSA + 1: 200 ITS-X + 0.25 mM ascorbic acid + 10 mM Nicotinamide (cat. No. N0636, Sigma).

Basal Differentiation Media 3 (**BDM3**)—MCDB131 + 2.5 g/L NaHCO_3_ + 1X Glutamax + 25 mM final glucose + 2.0% FAF BSA +1:200 ITS-X + 10 μg/mL Heparin (cat. No. H3149, Sigma) + 10 μM ZnSO_4_ (cat. No. Z0251, Sigma) + 0.25 mM ascorbic acid + 1X penicillin/streptomycin (cat. No. 15140148, Thermo Fisher).

Basal Differentiation Media 3.1 (**BDM3.1**)—MCDB131 + 2.5 g/L NaHCO_3_ + 1X Glutamax + 25 mM final glucose + 2.0% FAF BSA +1:200 ITS-X + 10 μg/mL Heparin + 10 μM ZnSO4 + 0.25 mM ascorbic acid + 1X penicillin/streptomycin + 1X MEM non-essential amino acids (cat. No. 11140050, NEAA, Thermo Fisher).

Basal Differentiation Media 4 (**BDM4**)—MCDB131 + 1.5 g/L NaHCO_3_ + 1X Glutamax + 8 mM final glucose + 2.0% FAF BSA + 10 μg/mL Heparin + 1 μM ZnSO_4_ + 1X penicillin/streptomycin + 1X MEM non-essential amino acids + 1X Trace elements A (cat No. MT99182CI, Corning) + 1X Trace elements B (cat. No. MT99175CI, Corning).

### 2.3. RNA Sequencing

RNA sequencing and subsequent bioinformatic analysis was done at SeqMatic (Fremont, CA, USA). Sequencing was done using Novaseq with dual index, 2 × 100 bp run parameter and S1 200 cycle reagent kit from Illumina (San Diego, CA, USA). Greater than 90% bases had a Quality (Q) score ≥ 30. Raw reads were trimmed of adapter sequences and low-quality bases using cutadapt. Clean reads were aligned to Human Genome Reference GRCh38 using HISAT2 resulting in >92% alignment to the genome. The software featureCounts was used to count the number of reads mapping to known genes as defined by Ensembl gene annotations. A time course study was performed using DESeq2. Normalized counts from DeSeq2 were log2 transformed and used for generating a heatmap showing differential expression of select genes during different stages (days).

### 2.4. Guide RNA Generation, CRISPR and Single Cell Cloning

sgRNA generation, hiPSC editing, and single cell cloning was done using the Cellartis iPSC rCas9 Electroporation and Single-Cell Cloning System (cat. No. 632643, Takara Bio) following manufacturer’s instructions. Briefly, a DNA template was generated by PCR using the Guide-it Scaffold template and a primer containing the T7 promoter sequence, the target sequence (20 base pairs upstream of the protospacer adjacent motif, NGG). The DNA template was in-vitro transcribed using Guide-it T7 polymerase mix to generate the sgRNA. The sgRNA was subsequently digested with DNase I and purified using the Guide-it IVT RNA clean-up kit and quantified using a QIAexpert system (cat. No. 9002340, Qiagen, Hilden, Germany). The sgRNA was then used to create a ribonucleoprotein (RNP) complex along with Guide-it recombinant CAS9 and was used for editing hiPSCs maintained in the DEF-CS 500 culture system by electroporation using a Neon transfection system (cat. No. MPK5000, Thermo Fisher). Electroporated hiPSCs were plated in a 48-well plate coated with COAT-1 from the DEF-CS 500 single cell cloning system (Takara) and allowed to recover for 4–5 days. Cells were harvested once they became confluent (but not dense) using TryPLE Select and single cell cloning was done using limiting dilution (0.5 cells/well of a 96-well plate). Colonies were expanded in 96 well-plates (cat. No. 2231730119, Eppendorf) for 10–14 days until ready for passaging. All wells were screened to ensure a single colony and these cells were passaged to a 48-well plate (cat. No. 2231723112, Eppendorf) using TrypLE select. Residual cells left in the 96-well plate after passaging were used for DNA isolation using QuickExtract DNA extraction solution (cat. No. QE09050, Lucigen, Middleton, WI, USA) and the DNA was screened for successful edits using Sanger sequencing. The clonal cell lines identified to contain the desired edits were expanded and frozen.

### 2.5. RNA Isolation and Quantitative Real-Time PCR

Total RNA was isolated using RNeasy plus micro kit (cat. No. 74034, Qiagen) and quantified by nanodrop (Thermo Fisher). 2 μg of total RNA was used for cDNA conversion using the high-capacity RNA-to-cDNA kit (cat. No. 4387406, Thermo Fisher). Real-time PCR was done using TaqMan probes (Thermo Fisher) for *KCNQ1*, *CDKN1C*, *IGF2*, *SLC22A18* and *KCNQ1OT1* (Data for Figure 3). For all others, real-time PCR was done using gene specific primers (Table S1), and PowerUP SYBR green master mix (cat No. A25777, Thermo Fisher). A dissociation curve analysis was done to ensure amplification of a single product. Expression levels were normalized to the expression of *TBP,* and relative expression was analyzed using 2^–∆∆Ct^ method. Results were compared using an unpaired *t*-test.

### 2.6. Copy Number Assay

Custom TaqMan copy number assays (Thermo Fisher) were designed to detect potential deletions in the CRISPR edited regions (Table S2). Genomic DNA samples were all normalized to 5 ng/μL. The custom copy number assays were run simultaneously with a copy number reference assay (either TaqMan Copy Number Reference Assay, human, TERT or RNaseP) in a duplex real-time polymerase chain reaction. The real-time PCR results were then analyzed using CopyCaller software v2.0 (Thermo Fisher).

### 2.7. Bisulphite Sequencing

500 ng of genomic DNA was bisulfite converted for methylation analysis using the EpiJET bisulfite conversion kit (cat No. K1461, Thermo Fisher) following manufacturer’s instructions. The bisulfite converted DNA was used to amplify regions of the *KCNQ1OT1* promoter using the following target specific primers: *FR1-F—TAGGATTTTGTTGAGGAGTTTTTTGGAG, FR1-R—AAACCAATCTAAACCCRAATAACATCAA, FR2-F—GAGTTGGAGATAYGGGTTAGTTTTTTG, FR2-R—CACAACAATAAAAACTTCAAAACATCCC.* The amplicons were then used for TA cloning and subsequent transformations using TOPO TA Cloning Kit for Sequencing and One Shot TOP10 Chemically Competent *E. coli* (cat No. K457501, Thermo Fisher). Twenty-four bacterial colonies were then selected for colony PCR and sequencing using either the T3 or T7 primers. Cloned amplicons were analyzed to identify methylated CpG sites (methylated cytosines will not be converted to thymine following bisulfite treatment). Methylation at each CpG site was calculated as the percentage of bacterial colony with a cytosine residue at that site. For each fragment, methylation was calculated as the average methylation across the CpG sites (FR1, 23 CpG sites, chr11:2699936-2700124 and FR2, 26 CpG sites, chr11: 2700337-2700613).

### 2.8. Genomic Stability

hiPSCs from the last passage prior to freezing were screened for genomic abnormalities by G-band karyotyping at WiCell (Madison, WI, USA). Only frozen cells from this passage with no clonal abnormality was used in further experiments.

### 2.9. Pluripotency and Differentiation Potential

Pluripotency and differentiation potential (directed differentiation towards pancreatic progenitors) was assessed by flow cytometry for stage specific markers (TRA-160 and SSEA4 for pluripotency, CXCR4 and CD117 for definitive endoderm and, PDX1 and NKX6-1 for pancreatic progenitors). RNA from the pluripotent stage and definitive endoderm stage was also used for hPSC scorecard assays done using TaqMan hPSC scorecard panel (cat. No. A15870, Thermo Fisher). The hPSC scorecard panel measures gene expression of a set of markers specific for pluripotency and the three germ layers (ectoderm, endoderm, and mesoderm) and compares this expression to that measured in undifferentiated established human embryonic stem cells in the form of an algorithmic score. The hPSC scorecard data was analyzed using the hPSC scorecard analysis software v 1.3 (Thermo Fisher, https://www.thermofisher.com/us/en/home/life-science/stem-cell-research/taqman-hpsc-scorecard-panel/scorecard-software.html (accessed on 29 September 2021)).

### 2.10. Off-Target Effect Analysis

CCTop—CRISPR/Cas9 target online predictor tool (https://cctop.cos.uni-heidelberg.de:8043/ (accessed on July 7 2019)) was used to identify the top 10 predicted off-target regions for each guide RNA that led to the successful generation and establishment of edited isogenic hiPSCs (sg2, sg3 and sg5, Table S3). The predicted off-target regions were Sanger sequenced and compared to the sequence from the parental cell line to identify any off-target effect.

### 2.11. Flow Cytometry

For staining of surface antigens, cells were harvested using TrypLE select as a single cell suspension, washed twice with dPBS, and resuspended in stain buffer (cat. No. 554656, BD Biosciences, Franklin Lakes, NJ, USA). For each test, 1 × 10^6^ cells were then incubated with fluorophore conjugated primary antibodies or isotype control antibodies for 45 min at room temperature. The cells were then washed twice with stain buffer and resuspended in 500 uL of stain buffer and analyzed using Accuri C6 plus flow cytometer (BD Biosciences). For intracellular staining, cells were harvested as a single cell suspension using TrypLE select (Thermo Fisher) and washed with cold dPBS. One million cells were used for each test and the cells were stained using LIVE/DEAD Fixable Red stain (cat. No. L23102, Thermo Fisher) following manufacturer’s instructions. The cells were then washed once with cold dPBS and fixed using 4% paraformaldehyde (cat No. 28908, Thermo Fisher) for 30 min on ice. The cells were then washed once with PBS and incubated in blocking and permeabilization buffer (PBS + 0.1% Triton X-100 + 5% donkey serum) for 1hr at 4 °C. The blocking buffer was removed, and cells were incubated in primary antibody or isotype control antibody diluted in blocking buffer overnight at 4 °C. Cells were then washed twice in blocking buffer and incubated in fluorophore conjugated secondary antibody diluted in blocking buffer for 2 h at 4 °C. Cells were washed 3 times in sorting buffer (PBS + 0.5% BSA) and resuspended in 500 uL sorting buffer, filtered using Flowmi cell strainers (cat No. H13680-0070, SP Bel-Art, Wayne, NJ, USA) and analyzed using Accuri C6 plus flow cytometer. Data were analyzed using Flowjo software v 10.6.1 (BD Biosciences). Dead cells were excluded during analysis and isotype control antibodies were used to determine gating. Gating strategy is shown in Figure S4. List of antibodies and isotype controls is given in Table S4.

### 2.12. Immunocytochemistry

For adherent cultures, cells were washed twice with PBS and fixed using 4% paraformaldehyde for 20 min at room temperature. Fixed cells were then washed twice with PBS and once with wash buffer (PBS + 0.1% BSA) and incubated in permeabilization and blocking buffer (PBS + 0.3% triton-x-100 + 10% normal serum from the host animal for the secondary antibody) for 45 min at room temperature. Cells were then incubated overnight at 4 °C with diluted primary antibody (Table S4) (dilution buffer- PBS + 1% BSA + 0.3% triton-x-100, 0.01% sodium azide + 1% normal serum from the host animal for the secondary antibody). The cells were washed three times with wash buffer and incubated with secondary antibody diluted in dilution buffer for 1hr protected from light. Secondary antibody was removed by washing three times with wash buffer and cells were counterstained using NucBlue fixed cell ready probe (cat. No. R37606, Thermo Fisher). For immunostaining of 3D clusters, the clusters were dissociated into single cells using TrypLE select. Cells were then deposited as a monolayer on Shandon cytoslides (cat. No. 5991056, Fisher Scientific) using a cytospin 4 centrifuge (cat. No. A78300003, Fisher Scientific). Slides were fixed, permeabilized and stained using primary and secondary antibodies (Table S4) as described above. The slides were counterstained using NucBlue fixed cell ready probe and mounted and cured using prolong gold antifade mountant (Thermo Fisher) before imaging. All images were captured using an EVOS FL cell imaging system (cat. No. AMF4300, Thermo Fisher) with built in image acquisition software.

### 2.13. Glucose Stimulated Insulin Secretion Assay

For static glucose stimulated insulin secretion assay, 25–30 islet-like clusters were rinsed twice using KRB buffer (128 mM NaCl + 5 mM KCl + 2.7 mM CaCl_2_ + 1.2 mM MgSO_4_ + 1 mM Na_2_HPO_4_ + 1.2 mM KH_2_PO_4_ + 5 mM NaHCO_3_ + 10 mM HEPES + 0.1% BSA, pH 7.4 with NaOH) and incubated in KRB buffer containing 2 mM glucose for 1hr for equilibration. The KRB buffer was replaced with fresh 2 mM glucose KRB and incubated for 1hr. The buffer was saved, and the clusters were washed once with KRB buffer and then incubated in 20 mM glucose KRB for 1hr. The buffer was retained, and the clusters were washed once with KRB buffer and then incubated in 30 mM KCl for 30 min. The buffer was retained, and the clusters were placed in acidified ethanol (75% ethanol + 1.5% HCl + 0.1% triton-X-100) and stored at −20 °C for 72 h. with vortexing every 24 h. Extracted insulin was collected as a supernatant by a brief centrifugation and used to normalize insulin secretions. The retained buffer was then used to quantify insulin secretion using Human Insulin Elisa (cat. No. 10-1113-01, Mercodia, Winston Salem, NC, USA).

## 3. Results

### 3.1. Differences in Gene Expression and Imprinting Status of KCNQ1, CDKN1C and TRPM5 during Pancreatic Beta-like Cell Differentiation from American Indian hiPSCs

**Figure 1:**
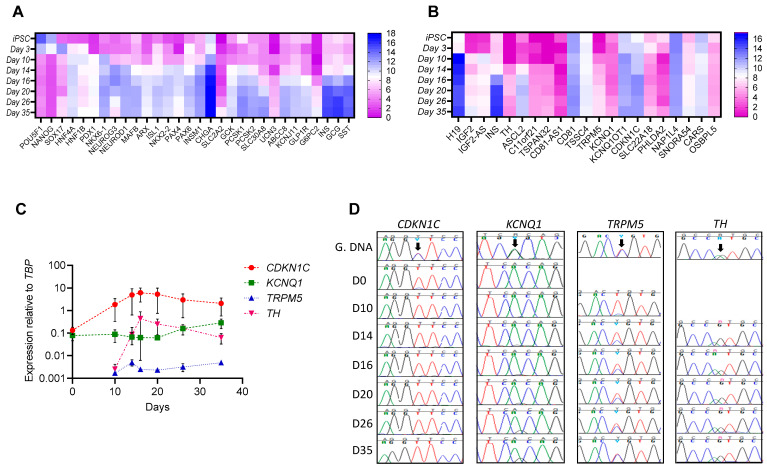
Gene expression and imprinting at the *KCNQ1* locus during beta-like cell development from hiPSCs. (**A**,**B**). Heatmap showing average deseq2 normalized counts from RNA sequencing of different stages of beta-like cell differentiation from three different American Indian hiPSCs for (**A**) selected stage specific differentiation markers and (**B**) genes in a 1.1 mb region at the *KCNQ1* locus. (**C**). Expression of *KCNQ1*, *CDKN1C*, *TRPM5* and *TH* during different stages of beta-like cell differentiation relative to the expression of *TBP* (housekeeping gene) from the same stage (*n* = 2). (**D**). Chromatograms showing a heterozygous coding SNP (arrow) in *KCNQ1*, *CDKN1C*, *TRPM5* and *TH* in genomic DNA (G.DNA) from hiPSCs and either monoallelic or biallelic expression (as seen by either one peak or two peak) in cDNA from different stages of differentiation. D0—iPSCs, D10—Pancreatic progenitors, D14 and D16—Endocrine progenitors, D20—immature beta-like cells, D26 and D35—mature beta-like cells.

hiPSCs from 3 different American Indian donors, whose DNA had been previously densely genotyped, were differentiated into pancreatic beta-like cells (Differentiation Protocol 1). Stage specific upregulation/downregulation in expression of genes important for pluripotency and pancreatic-beta cell development and function confirmed proper differentiation (Figure 1A). Profiling of expression levels for genes located in a 1.1 Mb region surrounding *KCNQ1* during different days of differentiation showed that several genes, including *H19*, *IGF2*, *IGF2-AS*, *INS*, *TH*, *ASCL2*, *KCNQ1OT1*, *CDKN1C* and *SNORA54* were dynamically regulated with peak expression seen during different stages of differentiation (Figure 1B and Table S5). In contrast, *KCNQ1* expression was consistent during all stages of differentiation (Figure 1B, C). Previously identified heterozygous exonic variants in *KCNQ1*, *CDKN1C* and *TRPM5* (which is known regulate glucose stimulated insulin secretion) were used to assess mRNA expression from both chromosomes during each stage of differentiation to identify gene-specific imprinting. *KCNQ1OT1* was not assessed since there were no heterozygous exonic variants in this gene. Expression of these 3 genes, as well as the nearby non-imprinted gene *TH* which served as a control, was verified using real-time PCR (Figure 1C) and Sanger sequencing of the cDNA was used to determine whether the genes were bi-allelically or mono-allelically expressed during each stage of differentiation (Figure 1D). Sequencing of the hiPSC genomic DNA confirmed the heterozygous state of the selected exonic SNPs for all four genes. *CDKN1C* had mono-allelic expression during all stages of differentiation. *KCNQ1* had mono-allelic expression during the iPSC stage (day 0), and early stages of differentiation while there was a gradual loss of imprinting in the immature beta-like cell stage (day 20) and mature beta-like cell stage (day 26 and day 35, Figure 1D). *TRPM5* was not detected in hiPSCs and started losing imprinting during the pancreatic progenitor stage and bi-allelic expression was seen during later stages. As expected, bi-allelic expression was found during all stages for the non-imprinted *TH* gene.

### 3.2. Generation of Genome-Edited Isogenic hiPSCs

#### 3.2.1. SNP Selection and Allele-Specific Guide RNA Design for CRISPR/CAS9 Editing at the *KCNQ1* Locus

**Figure 2:**
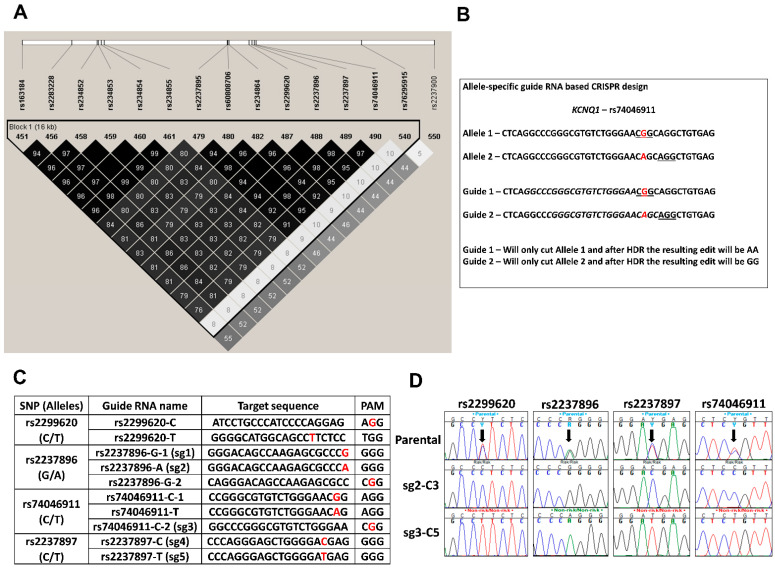
Experimental design and generation of genome-edited isogenic hiPSCs with edits at multiple SNPs. (**A**). Linkage disequilibrium at the T2D associated *KCNQ1* locus in Southwestern American Indians. Selected SNPs in strong LD with the T2D associated lead SNP (rs2299620) at the *KCNQ1* locus identified using whole-genome sequencing data from 296 American Indian subjects. (**B**). A representative example showing allele-specific guide RNA design to target the T2D associated rs74046911 SNP (highlighted in red) at the *KCNQ1* locus. DNA repair following CRIPSR will lead to loss of heterozygosity at nearby SNPs as well. Red—SNP, underline—PAM site, Italics—20 bp target sequence upstream of the PAM site. (**C**). Allele-specific Guide RNA design showing the targeted SNPs, target sequence (protospacer) and the PAM site. SNP site is indicated in red. (**D**). Generation of isogenic cells lines using allele specific CRISPR sgRNAs. Chromatograms showing successful edits at the four putative functional SNPs (arrows) from two representative clonal cell lines compared to the parental cell line.

The lead SNP, rs2299620, at the *KCNQ1* locus is in a region of very high linkage disequilibrium (Figure 2A). Preliminary data from our prior in-vitro studies provided evidence that a region containing the lead SNP and three other SNPs rs2237896, rs2237897 and rs74046911 may have a functional effect [25]. This finding is supported by ENCODE and Islet regulome databases which map putative strong enhancers to these SNPs with multiple epigenetic marks and transcription factor binding sites. We hypothesized that targeting this region using CRISPR and allele-specific guide RNAs (sgRNAs) in an hiPS cell line heterozygous for these variants and subsequent single cell cloning will generate isogenic hiPSCs that will differ at multiple SNP sites. Utilizing allele-specific sgRNA eliminates the need for donor DNA as a template for homology directed repair (HDR), and instead introduces a double strand break in only one chromosome at the specific site of interest such that the intact chromosome containing the other allele is used as the template, thereby changing the heterozygous SNP to a homozygous SNP as well as leading to edits at nearby SNPs (Figure 2B). To validate this technique and identify the most robust sgRNA sequences to target the 4 SNPs, 10 sgRNAs were designed such that the SNP mapped within the PAM site (protospacer adjacent motif; a 3 bp DNA sequence that follows the DNA region targeted for cleavage by spCas9) or within 6 bp of the PAM site (Figure 2C). These sgRNAs were tested for efficiency (data not shown) and analysis of the resulting edits by Sanger sequencing led to the selection of sgRNAs rs2237896-G-1 (sg1), rs2237896-A (sg2), rs74046911-C-2 (sg3), rs2237897-C (sg4) and rs2237897-T (sg5) for further experiments.

#### 3.2.2. Generation of Isogenic Cells Lines Using Allele Specific CRISPR sgRNAs

An American Indian hiPSC line heterozygous for the above variants was nucleofected with the RNP complex containing one of the selected sgRNAs and after subsequent recovery and expansion, single cell cloning was performed. A total of 490 single cell colonies were screened for the desired edits; among the 115 which were subsequently expanded and sequenced across the 4 targeted SNPs, 23 had no edits (i.e., all four SNPs were still heterozygous) and 71 had an indel at the CRISPR site. The remaining 21 cell lines had evidence of editing by HDR (Figure 2D, representative image showing two edited clonal cell lines). The genotypes of the 4 SNPs in all of the 21 edited cell lines were in accordance with the allele-specific sgRNA used in nucleofection.

### 3.3. Characterization of Genome-Edited Isogenic hiPSCs

#### 3.3.1. Extended Genotyping and Identification of Large Deletions in the Established Isogenic Cell Lines

**Figure 3:**
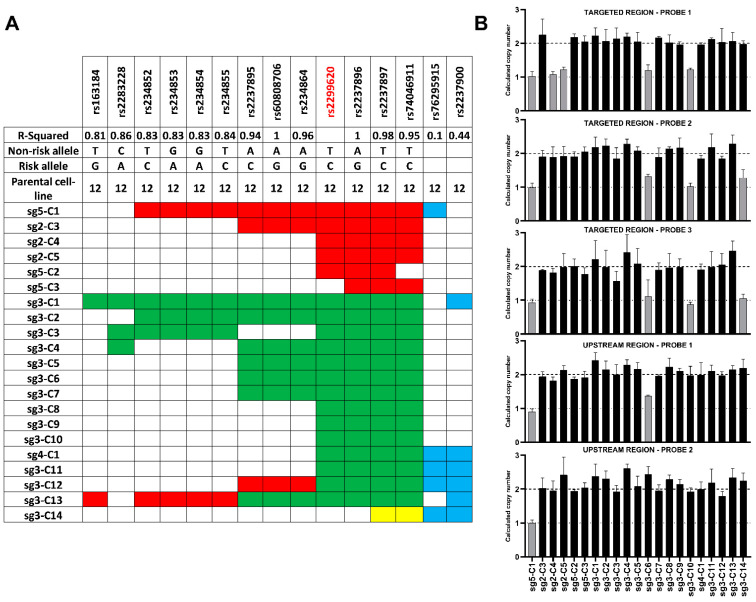
Extended genotyping of the clonal cell lines and identification of CRISPR induced large deletions. (**A**). Extended genotyping of the established clonal cell lines by sequencing to identify stretch of homozygosity. Parental cell line was heterozygous (12) for all of the SNPs. Red—homozygous for risk allele, green—homozygous for the non-risk allele, no fill—heterozygous (no edit), blue—homozygosity outside the LD block, yellow—deletion. The lead SNP is indicated in red. (**B**). CNV assay for identification of large deletions. Results of CNV assay using multiple probes in clonal cell lines. Results from the parental cell line was used as the calibrator for analysis. Y-axes represent the calculated copy number. Grey bars indicate a copy number of 1 suggestive of a hemizygous deletion. Assays were done in triplicate and the error bars indicate calculated copy number range.

The extent of homozygosity in the 21 cell lines with evidence of HDR was determined by genotyping (via sequencing) 15 SNPs spanning a 20 kb region (Figure 3A). The unedited parental cell line was heterozygous for all 15 SNPs whereas varying stretches of homozygosity was seen in the clonal cell lines (Figure 3A). In 7 of the 21 clonal cell lines, the edits were limited to the 4 SNPs in the putative functional region (2 clones, sg5-C2 and sg5-C3, with only 3 edited SNPs). Four clones had edits that covered all 7 SNPs in strong LD (r^2^ ≥ 0.95) with each other and the lead SNP (Figure 3A). Seven cell lines had evidence of HDR beyond the LD block (Figure 3A, blue blocks). The stretch of homozygosity extended as far as 11.5 kb upstream (Figure 3A, e.g., sg3-C1) and 8.9 kb downstream of the targeted site (Figure 3A, e.g., sg3-C11).

CRISPR also leads to on-target large deletions and complex rearrangements in edited cells [26,27]. These deletions, when hemizygous, give the appearance of homozygosity when the edited region is sequenced due to primer limitations. To identify any clonal cell lines which carry a hemizygous deletion, we performed copy number variation (CNV) analyses using 3 different custom Taqman CNV probes that map to the targeted region and 2 custom CNV probes that map upstream of the targeted region. Using the unedited parental cell line as the 2 copies reference, 6 of the 21 cell lines had a predicted copy number of 1 for one or more CNV probes, indicating that large deletions had occurred in one of the chromosomes (Figure 3B). We also observed complex repair events that resulted in 2 clonal cell lines (sg3-C12 and C13, Figure 3A) homozygous for the risk allele (red squares) and homozygous for the non-risk alleles at different SNP sites.

#### 3.3.2. Identification of Clonal Cell Lines with Hypomethylation at the *KCNQ1OT1* Promoter Region Resulting in Downregulation of *KCNQ1* Expression

**Figure 4:**
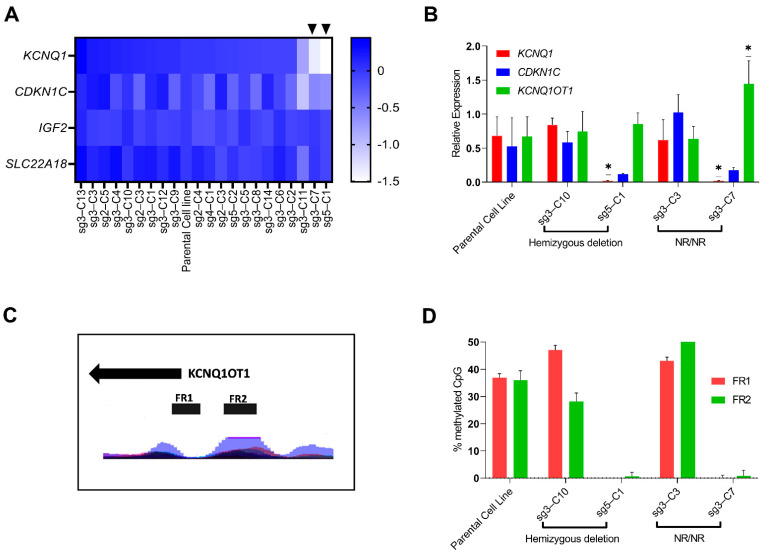
Identification of clonal cell lines with hypomethylation at the *KCNQ1OT1* promoter region resulting in downregulation of *KCNQ1*. (**A**). Heat map showing expression (logFC) of *KCNQ1*, *CDKN1C*, *IGF2* and *SLC22A18* in the clonal cell lines relative to expression in the parental cell line. Data is using RNA from a single passage. Arrows heads show cell lines with downregulation of *KCNQ1*. (**B**). Relative expression of *KCNQ1*, *CDKN1C* and *KCNQ1OT1* in selected clonal cell lines. Clones sg3-C10 and sg5-C1 had hemizygous deletions in different alleles and of different size and clones sg3-C3 and sg3-C7 had edits resulting in homozygous non-risk alleles at the *KCNQ1* locus. Relative expression was normalized to the expression of TBP. RNA from three different passages were used for the experiment. Error bars show SD and results were compared using *t*-test, * *p* < 0.05. (**C**). Layered H3K27Ac marks in 7 cell lines from ENCODE in the region captured by FR1 and FR2 used for bisulfite sequencing to analyze methylation at the *KCNQ1OT1* promoter. (**D**). Average methylation across 49 CpG sites in the *KCNQ1OT1* promoter region assessed by bisulphite sequencing. Two different regions were assessed, FR1 (23 CpG sites) and FR2 (26 CpG sites). Error bars show SD.

Regulation of gene expression at the *KCNQ1* region is affected by methylation at the *KCNQ1OT1* promoter, which is maternally methylated and paternally unmethylated. To test whether the process of single cell cloning following CRISPR could result in the expansion of clonal cell lines hypomethylated in this region, which in turn may affect gene expression irrespective of the genotype, we compared expression of *KCNQ1*, *CDKN1C* and *SLC22A18* (genes regulated by the imprinting control region (ICR) in intron 10 of *KCNQ1*) and *IGF2* (regulated by the ICR 5′ of *H19*) by real-time PCR in parental vs. established clonal cell lines. This identified downregulation of *KCNQ1* and *CDKN1C* expression in 2 clonal cell lines while no difference was seen in the expression of *SLC22A18* and *IGF2* (Figure 4A). This finding was verified by analyzing gene expression of *KCNQ1*, *CDKN1C* and *KCNQ1OT1* in 5 clonal cell lines and the parental cell line from three different passages which identified significant downregulation of *KCNQ1* expression and upregulation of *KCNQ1OT1* expression not entirely attributable to their genotypes (Figure 4B).

Bisulphite sequencing of 49 CpG sites across the promoter of *KCNQ1OT1* (FR1, chr11:2699936-2700124 and FR2, chr11: 2700337-2700613, Figure 4C) confirmed that the difference in expression was due to differences in methylation at the *KCNQ1OT1* promoter (Figure 4D). In the parental and clonal cell lines (sg3-C10 and sg3-C3) with no differences in the expression of *KCNQ1* and *KCNQ1OT1,* the average methylation across the 49 CpG sites were 36.4%, 37.6% and 46.5% respectively. In contrast, in clonal cell lines (sg5-C1 and sg3-C7) with significant downregulation of *KCNQ1* and upregulation of *KCNQ1OT1* expression compared to the parental cell line, the average methylation ranged from 0.28–0.53% (Figure 4D).

#### 3.3.3. Genomic Stability, CRISPR-cas9 Off-Target Effects, and Pluripotency in the Established Isogenic Cell Lines

The parental and 12 clonal cell lines with no edits outside the LD block and no complex rearrangement were selected for further characterization by G-banded karyotyping. This identified two cell lines with genomic abnormalities (sg2-C5 and sg3-C6). Seven of the remaining clonal cell lines with normal karyotypes, no large deletions, and *KCNQ1* expression similar to the parental cell line were further examined for CRISPR off-target effects. The top 10 predicted off-target regions were sequenced for each of the 7 clonal cell lines, only 1 clone had an off-target effect located in an intergenic region (Table 1). These 7 clonal cell lines were further assessed for pluripotency using an hPSC scorecard panel as described in the methods. The algorithmic score in all clonal cell lines were comparable to the scores in established human embryonic stem cells for pluripotency (Table 1).

### 3.4. Functional Characterization of a Clonal Cell Line with a CRISPR-Induced Hemizygous Deletion in KCNQ1

#### 3.4.1. A 2.3 kb Deletion in *KCNQ1* That Spans the 4 Putative Functional SNPs Affects Gene Expression of *CDKN1C* and *H19*

**Figure 5:**
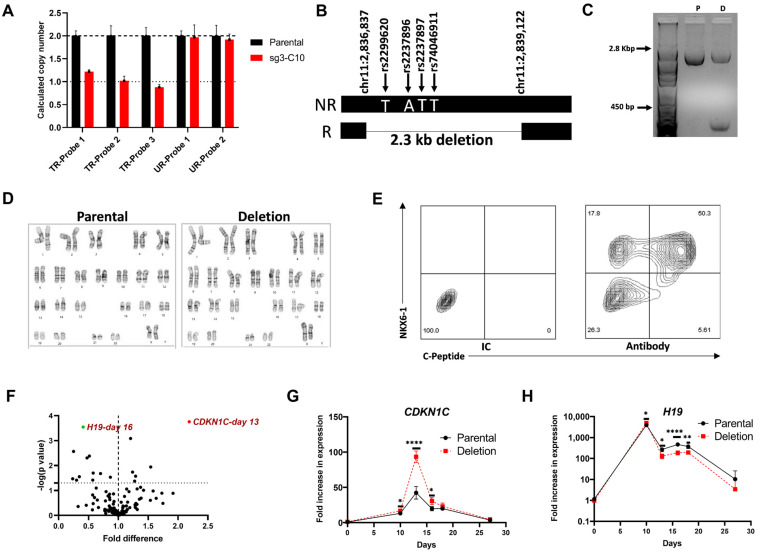
Stage specific differential expression of *CDKN1C* and *H19* in cells with a 2.3 kb hemizygous deletion in *KCNQ1* intron 15 that spans the four putative functional SNPs. (**A**–**D**). Characterization of a clonal cell line with a 2.3 kb hemizygous deletion at the *KCNQ1* locus. (**A**). Copy number assay (CNV) showing hemizygous deletion in sg3-C10 (deletion cell line). TR probes—CNV probes mapping to the CRISPR targeted region. UR probes—CNV probes mapping to the upstream region. Data shown is part of Figure 3B. (**B**). Figure showing the 2.3 kb deletion in the allele with the risk haplotype for the four putative functional SNPs at the *KCNQ1* locus. (**C**). Confirmation of the hemizygous deletion by agarose gel electrophoresis of the PCR amplicon generated by long PCR using primers spanning the deletion. P = parental cell line, D = deletion cell line (sg3-C10). (**D**). Karyotyping by G-banding of the parental and deletion cell line. (**E**). Representative flow cytometry data of islet-like cells generated by differentiating the deletion cell line using differentiation protocol 2 showing staining for INS (C-peptide) and the beta cell transcription factor NKX6-1. IC—isotype control. Data also shown in Figure S4. (**F**–**H**). Stage specific differential expression of *CDKN1C* and *H19* during islet-like cell differentiation from hiPSCs. (**F**). Statistical significance and fold difference in the expression of 20 genes at the *KCNQ1* locus in the cells with the deletion relative to the parental cells during days 0, 10, 13, 16, 18 and 27 of differentiation. The *x*-axis shows the fold difference in expression and the *y*-axis show the -log(*p*-value). The dashed horizontal line indicates a *p* = 0.05. Results are from four independent differentiations and expression was calculated relative to expression in the parental cells from experiment 1 from the respective days. Results were compared using *t*-test. (**G**,**H**). Fold change in the expression of *CDKN1C* and *H19* during differentiation of the deletion cell line and the parental cell line to pancreatic islet-like cells relative to the expression in hiPSCs. Results are from four independent differentiations; the error bars show SD and results were compared using *t*-test. Expression was calculated relative to expression in parental hiPSC from experiment 1. * *p* < 0.05, ** *p* < 0.01, **** *p* < 0.0005.

To confirm that the region containing the lead T2D-associated SNP and 3 neighboring SNPs (rs2299260, rs2237896, rs2237897 and rs74046911) is functionally important, we selected a clonal cell line (sg3-C10, from now on called the deletion cell line) with a CRISPR-induced hemizygous deletion (Figure 5A) and assessed its role in pancreatic islet development. Using a combination of long PCR and nested sequencing primers, we determined that the deletion (chr11:2836837-chr11:2839122) is approximately 2.3 kb, spans the 4 SNPs and is located in the chromosome carrying the diabetes risk haplotype (Figure 5B,C). The deletion carrying clonal cell line has a normal karyotype (Figure 5D), no CRISPR off-target edits and methylation at the *KCNQ1OT1* promoter is comparable to the parental cell line (Figure 4D). Pluripotency and differentiation potential of the parental and deletion cell lines were confirmed using the hPSC scorecard assay (Figure S5), and flow cytometry for pluripotency markers (SSEA4 and TRA-1-60), definitive endoderm markers (CXCR4 and CD117), and pancreatic progenitor markers (PDX1 and NKX6-1) following directed differentiation (Figure S5). Similar differentiation efficiencies were observed for both cell lines.

The parental and deletion cell lines were differentiated to generate pancreatic islet-like cells (Figure 5E) using the optimized seven-stage differentiation protocol. RNA was isolated from both cell lines and relative expression of genes in the 1.1 Mb region surrounding *KCNQ1* in the deletion cell line was compared to parental cell line at specific differentiation stages (day 0—iPSC, day 10—pancreatic progenitors, day 13 and day 16—endocrine progenitors, day 18—immature islet-like cells and day 27—mature islet-like cells). This identified 2 genes, *CDKN1C* and *H19*, as significantly differentially expressed between the 2 cell lines on day 13 (early endocrine progenitors) and day 16 (late endocrine progenitors), respectively (Figure 5F and Table S2). Comparison of expression changes over time (temporally) during differentiation relative to the parental hiPSC (D0) showed a similar pattern; there was an approximately 2X greater fold change in *CDKN1C* expression on day 13 and 50% lower fold change of *H19* expression on day 16 in the cells with the deletion (Figure 5G,H). In contrast, there was no significant difference in *KCNQ1* expression during any stage of differentiation between the two cell lines (Figure S6). The gene expression patterns during differentiation for the other genes are shown in Figure S6. Differentiation efficiency was monitored by analyzing the gene expression of stage specific differentiation markers and was found to be comparable for the parental and deletion cell lines (Figure S7).

#### 3.4.2. Differential Expression of INS in Day 27 Islet-like Cells Derived from the hiPSC with the 2.3 kb Hemizygous Deletion

**Figure 6:**
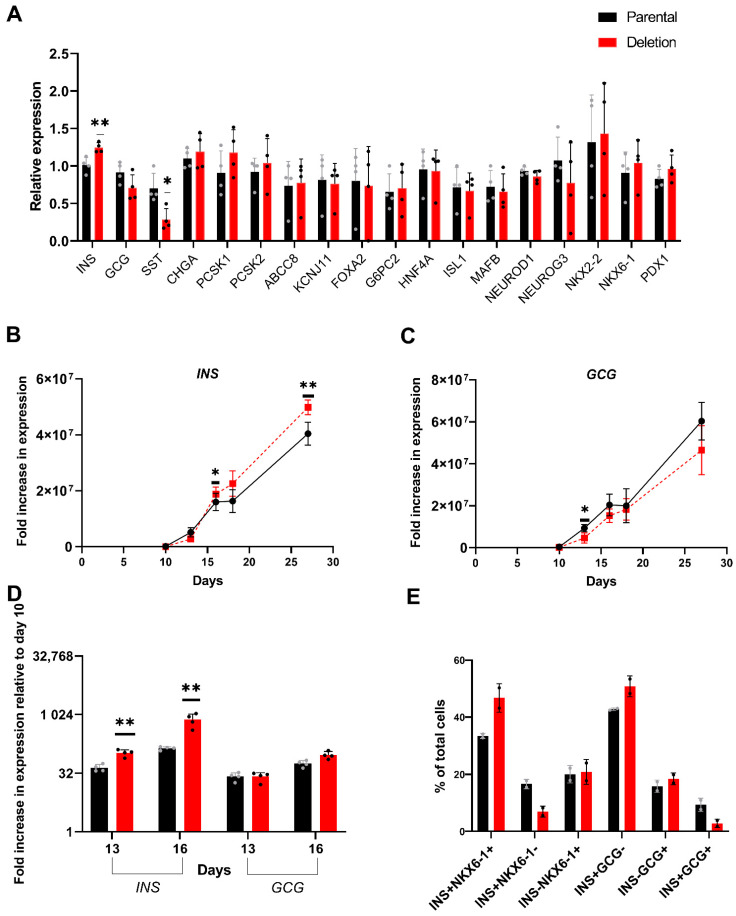
Stage specific differential increase in *INS* gene expression in cells with the 2.3 kb hemizygous deletion (**A**). Expression of islet specific genes in day 27 islet-like cells generated from hiPSC with the deletion relative to expression in day 27 islet-like cells generated from the parental hiPSC. Results are from four independent differentiations and the error bars show SD. Expression was calculated relative to expression in islet-like cells generated from the parental hiPSC in experiment 1 and results were compared using t-test. (**B**,**C**). Fold increase in the expression of *INS* and *GCG* expression during differentiation of the deletion iPS cell line and the parental iPS cell line towards pancreatic islet-like cells relative to the expression in hiPSCs. Results are from four independent differentiations and the error bars show SD. Expression was calculated relative to expression in parental hiPSC from experiment 1 and results were compared using *t*-test. (**D**). Fold increase in *INS* and *GCG* expression during the endocrine progenitor stage (day 13 and 16) relative to expression in pancreatic progenitors (day 10) from the parental and deletion cell lines. (**E**). Flow cytometry analysis of day 27 islet-like cells. Results are from two independent differentiations and error bars show SD. * *p* < 0.05, ** *p* < 0.01.

The effect of the 2.3 kb hemizygous deletion on expression of 18 genes important for islet development and function was examined in hiPSC derived mature islet-like cells (day 27). A nominal but significant (*p* = 0.009) increase in *INS*, which maps ~675 kb upstream of the *KCNQ1* T2D locus, and a modest decrease in *SST* (*p* = 0.02) gene expression was observed in the islet-like cells with the deletion (Figure 6A). There were no significant differences in expression of the other 16 genes, although there was a trend towards decreased expression for the alpha-cell gene *GCG* and increased expression for beta-cell specific genes *PDX1* and *NKX6-1* and insulin processing genes *PCSK1* and *PCSK2* in the islet-like cells with the deletion (Figure 6A). Further analysis of *INS* and *GCG* gene expression, the two main islet hormones, over the course of differentiation (Figure 6B,C) identified a relatively higher fold increase in expression of *INS* (113964-fold vs. 27821-fold, *p* = 0.0001) and *GCG* (356329-fold vs. 171513-fold, *p* = 0.007) in day 10 pancreatic progenitor cells generated using the parental cell line. However, endocrine commitment and robust increase in *INS* and *GCG* expressing cells during differentiation occurs only after the pancreatic progenitor stage. Analysis of *INS* and *GCG* expression during later stages of islet-like cell development relative to expression in pancreatic progenitors (day 10) showed that the fold increase in *INS* was significantly higher during the endocrine specification stage (day 11 to day 16) in the cells with the deletion. Relative to pancreatic progenitors (day 10), there was a 44.4-fold and 139.8-fold increase in *INS* expression during the endocrine progenitor stage (day 13 and day 16 respectively) in the parental cells, whereas there was a 106.7-fold and 763.3-fold increase in the cells with the deletion (*p* = 0.002 and *p* = 0.005, respectively, Figure 6D). In comparison, there was no significant difference in fold increase in the expression of *INS* during the islet maturation stage (day 27) relative to the endocrine progenitor stage (day 16). No significant differences in fold increase in *GCG* expression was seen on day 13 and only a nominal difference was seen on day 16 relative to day 10 (Figure 6D). This suggests that the difference in *INS* gene expression in day 27 islet-like cells shown in Figure 6A is due to differences in beta-like cell specification during the endocrine progenitor stages, which coincides with differences in *CDKN1C* and *H19* expression during this stage. Flow cytometry analysis also detected a higher number of functional (INS^+^/NKX6-1^+^) beta-like cells in day 27 islet-like cells generated from hiPSCs with the deletion (Figure 6E). There was no difference in the percentage of monohormonal GCG positive (INS^−^/GCG^+^) cells, whereas there were lower number of polyhormonal (INS^+^/GCG^+^) cells in the islet-like cells with the hemizygous deletion (Figure 6E).

## 4. Discussion

T2D is a common polygenic disease whose prevalence varies between different ethnic groups; among the hundreds of variants that associate with this disease, many are common to different populations with notable exceptions. For example, variation in *TCF7L2* has a strong effect on T2D in several ethnic groups except for Southwestern American Indians [28]. In contrast, variation in the imprinted *KCNQ1* gene has a particularly strong parent-of-origin effect for T2D in Southwestern American Indians [10,24]. Therefore, to fully understand how genetic variants at the *KCNQ1* locus influence the development of T2D in this group of American Indians, it is important to study the variants using cell lines with the appropriate genomic background. The current study details our efforts to generate American Indian specific isogenic hiPSCs for studying the effects of *KCNQ1* variation during differentiation to pancreatic islet-like cells. We show that these hiPSCs retain imprinting at the *KCNQ1* locus and that *CDKN1C* and *KCNQ1* had monoallelic expression at different stages of differentiation to pancreatic islet-like cells which is consistent with a previous report in fetal pancreas and adult islets [16]. Therefore, our American Indian hiPSC are an appropriate cell line for studying the T2D associated variants in the imprinted *KCNQ1* locus to understand their effect on the pathomechanism of T2D.

Most GWAS SNPs map to intronic or intergenic regions including the T2D associated variants in *KCNQ1*. These non-coding variants are often in high linkage disequilibrium with nearby SNPs and can exert their effects as a haplotype. Studying the molecular effects of a haplotype will require multiple edits in the same cells using CRISPR/CAS9. Using CRISPR to edit non-coding variants poses a particular challenge. During CRIPSR/CAS9 editing, donor DNA with the desired edits serves as a template to repair the CRISPR induced cleavage resulting in the insertion of the desired edits into the chromosome. The donor template usually contains a disrupted PAM site (NGG, the site recognized by SpCAS9) to prevent CAS9 from cleaving the edited DNA. If the GWAS variant being studied happens to disrupt the PAM site it can be edited by targeted CRISPR/CAS9 without unwanted cleavage. However, if the GWAS intronic variant is not in a PAM site, a second PAM-disrupting mutation must be introduced which could potentially confound interpretation of which variant caused an observed effect. We addressed this issue by not using donor DNA to insert the desired edits, but instead we used allele-specific guide RNAs targeting the region of interest and allowing homology directed repair to fix (edit) the cleaved region using the other intact chromosome as the template. Using this genome-editing strategy in a hiPSC heterozygous for the variants of interest, we were able to establish isogenic clonal cell lines with different combinations (haplotypes) of SNPs located in the LD block encompassing the putative functional variants with no CRISPR induced genomic abnormalities and proper methylation status. These established clones will be used in future studies to examine the effects of the T2D associated variants in *KCNQ1*.

Characterization of the isogenic hiPSCs identified clonal cell lines that were hypomethylated compared to the parental cell line. In any given cell line, individual cells can differ in their methylation status. Single cell cloning and expansion of a cell which is hypo- or hypermethylated compared to the parental cell line may result in a clonal cell line that differs in methylation status which in turn can affect gene expression. This is of particular concern when the target region is known to affected by methylation (e.g., *KCNQ1* locus). Therefore, studies using single cell cloning to derive isogenic hiPSCs for comparative analysis should ensure comparable methylation at the regulatory regions of the target loci. Our study also confirmed that CRISPR can cause undesired on-target mutagenesis (e.g., large deletions) which, when hemizygous, cannot be detected by routine sequencing. Therefore, careful screening using CNV probes targeting the edited region is important to ensure that homozygous cell lines are truly homozygous and are not actually hemizygous. In the current study, we identified a clone with a hemizygous deletion in intron 15 of *KCNQ1* which spans the four putative functional SNPs and utilized this aberrant clone to demonstrate the feasibility of using our edited isogenic hiPSC to study the effects of the *KCNQ1* variants on islet cell development and function. We show that the deletion effects the expression of *CDKN1C* and *H19*, with the strongest differences in expression seen during the endocrine progenitor stage. *CDKN1C*, which codes for the cell cycle inhibitor p57^kip2^, and *H19*, a long non-coding RNA, are known to inhibit and promote cell division, respectively [29,30,31]; and both of these genes have been implicated in regulation of pancreatic beta cell mass [30,32,33]. It has also been suggested that *CDKN1C* may be the effector gene at this T2D locus and that the SNPs may be exerting their effects during early pancreatic islet development [16]. Our results support these observations.

Our data also show that during the development of islets from hiPSCs, the expression of *CDKN1C* peaks during the endocrine progenitor stage and decreases thereafter. Studies of mouse islet development and maturation support this observation [34]. It has been proposed that the PDX1 expressing pancreatic progenitor cells that continue receiving notch signaling undergo self-renewal resulting in expansion of progenitor mass whereas the progenitor cells that do not receive notch signaling express NGN3 and p57, exit cell cycle, and undergo terminal differentiation [35]. Our data support that an increase in *CDKN1C* (p57) expression at this stage will favor exit of the endocrine progenitor cells from cell cycle resulting in terminal differentiation to a beta-cell fate. In endocrine progenitor cells with the hemizygous deletion, we observed a significant increase in *CDKN1C* expression concomitant with a modest increase in *INS* and *NKX6-1* (beta-cell specific transcription factor) gene expression. These results were supported by flow cytometry which showed that there was an increase in INS/NKX6-1 co-expression (indicative of functional beta-like cells) in the islets carrying the deletion. These observations indicate that the deletion of the *KCNQ1* region which contains that T2D risk haplotype affects beta-cell mass; therefore, it is possible that the T2D risk allele(s) decrease *CDKN1C* expression during the endocrine progenitor stage resulting in reduced pancreatic beta-cell mass.

## 5. Conclusions

In summary, we demonstrated that the intronic region in *KCNQ1* containing the T2D and insulin secretion associated variants is potentially important for islet development. However, the main limitation of our study is that the 2.3 kb deletion utilized in this study could include regulatory elements that regulate *CDKN1C* and *H19* expression independent of the SNPs. Therefore, future studies will use our established American Indian isogenic hiPSCs homozygous for either the T2D risk or non-risk haplotypes to examine the effects of the T2D risk alleles on pancreatic islet development and function and to detect any potential ethnic-specific pathomechanism underlying the development of T2D in American Indians.

## Figures and Tables

**Table 1:** Genomic Stability, CRISPR-cas9 off-target effects, and pluripotency in the established isogenic cell lines.

				hPSC Scorecard Scores (X)			
Cell line ID	Haplotype	Karyotype	Off-Target Edit	Pluripotency	Ectoderm	Mesoderm	Endoderm
Parental Cell line	MRRYRYY	46, XX	Reference	−0.2	−0.67	−0.73	−1.15
sg2-C3	CGGCGCC	46, XX	None detected	−0.5	−0.38	−0.6	−0.83
sg5-C2	MRRCGCY	46, XX	Intergenic Indel	−0.37	−0.62	−0.99	−1.22
sg5-C3	MRRYGCC	46, XX	None detected	−0.22	−0.54	−0.58	−1.14
sg3-C2	AAATATT	46, XX	None detected	0.08	−0.62	−0.72	−1.28
sg3-C5	AAATATT	46, XX	None detected	0.27	−0.56	−0.72	−1.18
sg3-C8	MRRTATT	46, XX	None detected	0.26	−0.49	−0.69	−1.05
sg3-C9	MRRTATT	46, XX	None detected	0.29	−0.62	−0.6	−1.28

Haplotype, karyotype, off-target edit and hPSC scorecard assay result in the established clonal cell lines and the parental cell line. Haplotypes refers to 7 SNPs in strong LD with the lead T2D variant at the *KCNQ1* locus; rs2237895 (C > A), rs60808706 (A > G), rs234864 (A > G), rs2299620 (T > C), rs2237896 (A > G), rs2237897 (T > C) and rs74046911 (T > C). One clonal cell line had an intronic indel in the IQCJ-SCHIP1 locus. hPSC scorecard assay results are shown as an algorithmic score derived using expression of pluripotency markers and the markers specific for the three germ layers. Scores (X) relative to the reference standard (expression in established human embryonic stem cell line) X > 0.5 (upregulated), −0.5 ≤ X ≤ 0.5 (comparable), X < −0.5 (downregulated).

## Data Availability

The RNA sequencing data used in Figure 1A,B are exempt from submission to public databases due to restrictions on genomic data generated in American Indians; however, these data are available upon request from the corresponding author. All other relevant data presented in this study are available within the article or supplementary material.

## Supplementary Materials

The following supporting information can be downloaded at: https://www.mdpi.com/article/10.3390/cells11091446/s1. Figure S1: Characterization of American Indian hiPSCs; Figure S2: Variable differentiation efficiency to generate pancreatic progenitors using differentiation protocol 1; Figure S3: An optimized seven-stage differentiation protocol (differentiation protocol 2) for generation of pancreatic-islet like cells from American Indian hiPSCs; Figure S4: Gating strategy used for flow cytometry analysis and representative flow cytometry data from different stages of differentiation; Figure S5: Pluripotency and differentiation potential of the parental and the deletion (sg3-C10) hiPSCs; Figure S6: Stage-specific expression of genes at the *KCNQ1* locus during different stages of differentiation from the parental hiPSC and deletion hiPSC; Figure S7: Stage specific expression of pancreatic islet differentiation markers during different stages of differentiation from the parental hiPSC and deletion hiPSC; Table S1: Gene specific primers used for real-time PCR; Table S2: Copy number assay design used for detecting large deletions; Table S3: Predicted off-target regions for guide RNAs used to generate the isogenic edited cell lines; Table S4: Antibodies used for flow cytometry and immunocytochemistry; Table S5: Deseq2 Normalized counts of genes at the *KCNQ1* locus at days of differentiation; Table S6; Expression of genes at the *KCNQ1* locus in the deletion cell line relative to the parental cell line during different days of differentiation; Table S7: Abbreviations.
