# Supplementary material for: Generation of Isogenic hiPSCs with Targeted Edits at Multiple Intronic SNPs to Study the Effects of the Type 2 Diabetes Associated KCNQ1 Locus in American Indians
Source: Cells. 2022 Apr 25;11(9):1446. doi: 10.3390/cells11091446 (PMC9102014; doi:10.3390/cells11091446)
Supplement: Supplementary file 1 [file cells-11-01446-s001.zip › Supplementary Figures S1-S7.pdf]

A

Sample Name	Self-renewal	Ectoderm	Mesoderm	Endoderm
hiPSC1 - iPSCs	-0.09	-0.17	-0.18	-0.97
hiPSC2 - iPSC	0.06	-0.15	-0.62	-0.69
hiPSC3 - iPSC	0.14	-0.71	-0.65	-1.05
hiPSC1 - Day 3	-3.19	-0.68	-0.45	2.04
hiPSC2 - Day 3	-6.00	0.53	0.35	1.96
hiPSC3 - Day 3	-2.96	-0.46	-0.81	2.15

Gene expression relative to the reference standard

Upregulated						Downregulated
x > 1.5	1.0 < x ≤ 1.5	0.5 < x ≤ 1.0	-0.5 ≤ x ≤ 0.5	-1.0 ≤ x < -0.5	-1.5 ≤ x < -1.0	x < -1.5

B

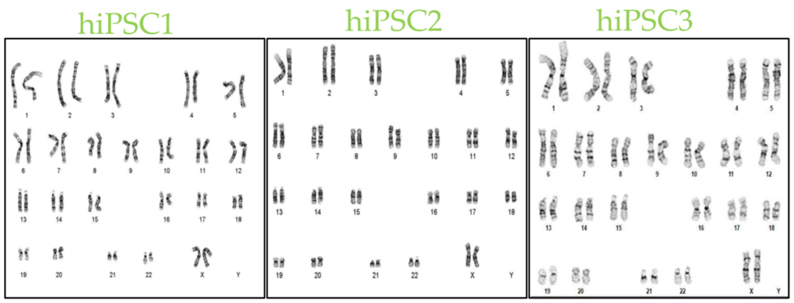

C

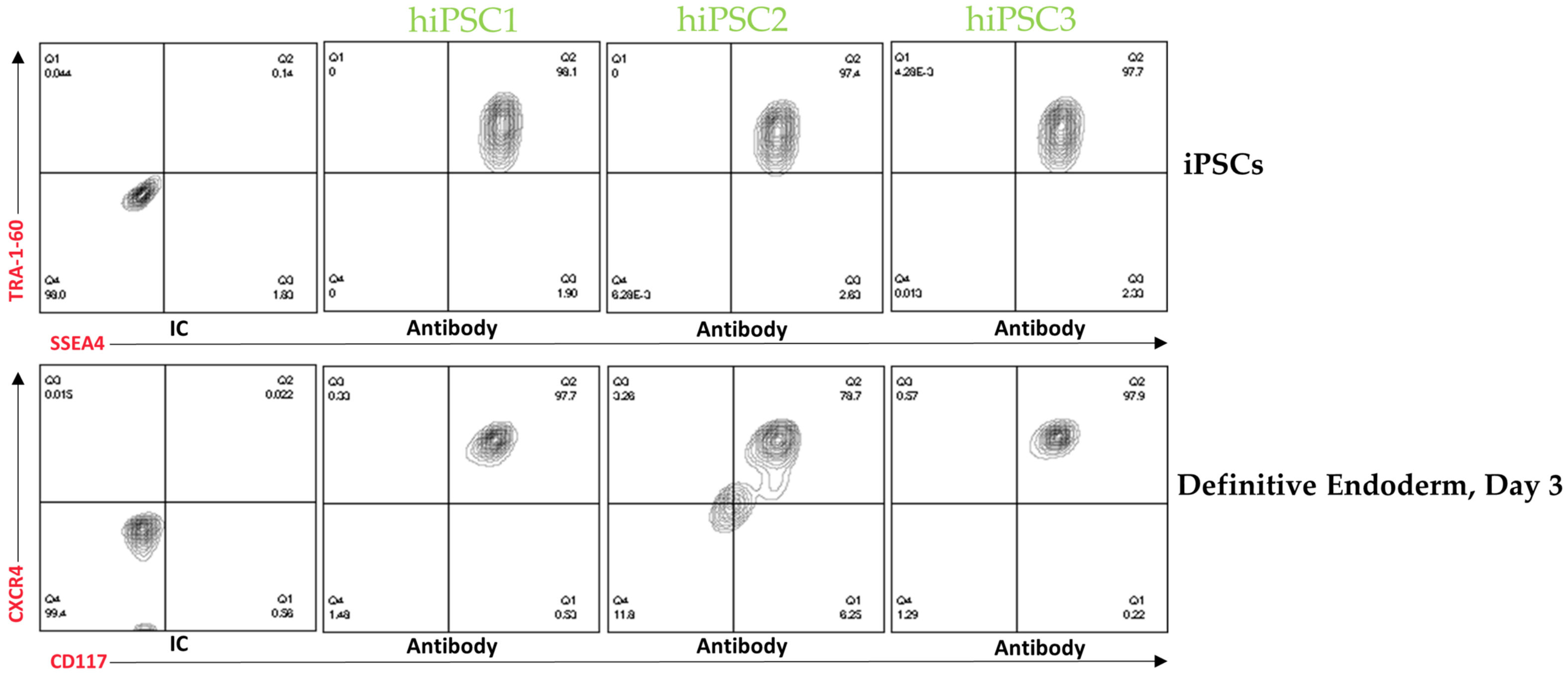

Figure S1. Characterization of American Indian hiPSCs. A. Algorithmic scores from hPSC scorecard assay using RNA from the iPSC stage and definitive endoderm stage (generated after three days of directed differentiation). Reference standard – established human embryonic stem cell lines. Data shows comparable expression of pluripotency markers during the iPSC stage whereas upregulation of endoderm markers and downregulation of pluripotency markers in the definitive endoderm stage relative to the reference standard. B. G-band karyotyping of the three hiPSCs. C. Flow cytometry analysis for TRA-1-60 and SSEA4 during the iPSC stage and for CXCR4 and CD117 after three days of directed differentiation towards definitive endoderm. IC-Isotype control.

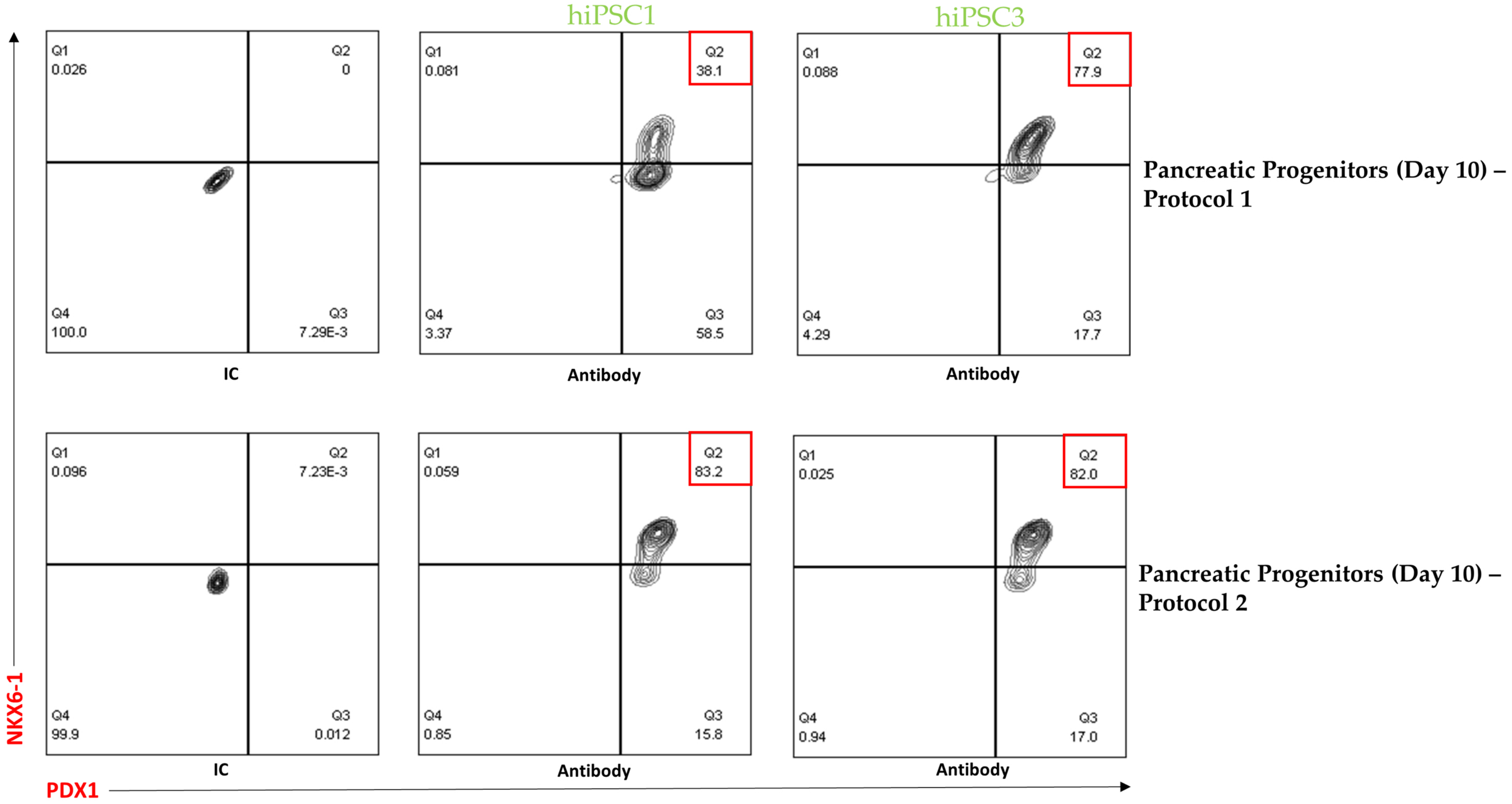

Figure S2. Variable differentiation efficiency to generate pancreatic progenitors using differentiation protocol 1. Data shows representative flow cytometry analysis of pancreatic progenitors generated after 10 days of directed differentiation using protocol 1 and protocol 2 from hiPSC1 and hiPSC3. Red box indicates percentage of cells co-expressing PDX1 and NKX6-1. Variable differentiation efficiency using protocol 1 was also seen with multiple isogenic clonal hiPSCs. IC- Isotype control.

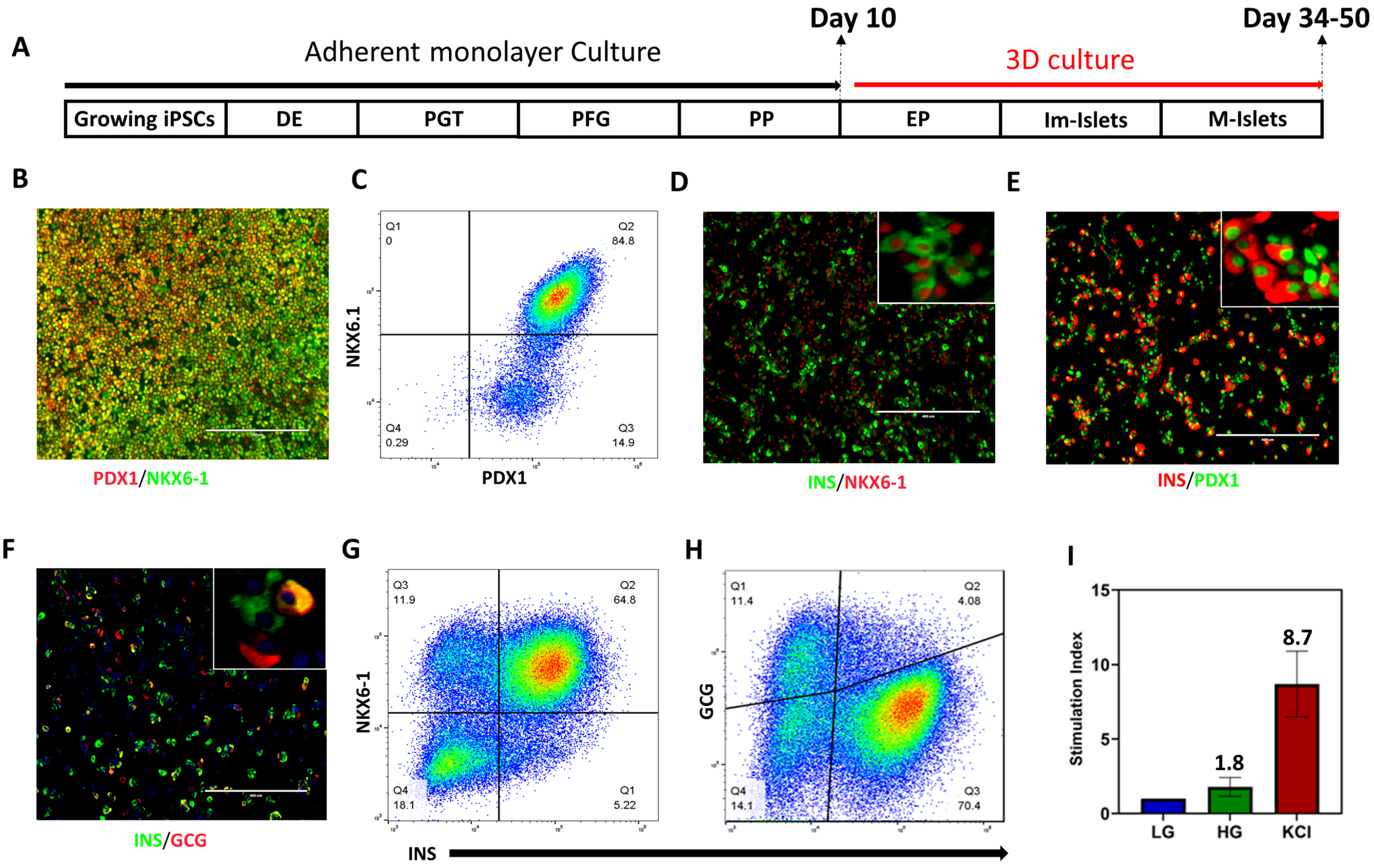

Figure S3. An optimized seven-stage differentiation protocol (differentiation protocol 2) for generation of pancreatic-islet like cells from American Indian hiPSCs. **A**. Overview of the optimized seven stage differential protocol used for generating islet-like cells from hiPSCs. DE – definitive endoderm (day 2), PGT – primitive gut tube cells (day 4), PFG – posterior foregut cells (day 6), PP – pancreatic progenitor cells (day 10), EP – endocrine progenitor cells (day 13), Im-Islet – immature islet-like cells (day 18), M-Islets – mature islet-like cells (day 27-50). **B-C**. Representative ICC image (B) and flow cytometry (C) showing staining for PDX1/NKX6-1 in day 10 pancreatic progenitors generated using differentiation protocol 2. Scale bar = 200um. **D-F**. Representative ICC image (scale bar = 400um) showing staining for INS and NKX6-1 (D), INS and PDX1 (E) and INS and GCG (F) in islet-like cells (Day 20) generated using differentiation protocol 2. Inserts represent scaled up section showing cells co-expressing INS and beta-cell transcription factors NKX6-1 (D) and PDX1 (E) and monohormonal INS and GCG positive cells as well a polyhormonal cell expressing both INS and GCG (F) **G-H**. Flow cytometry analysis of islet-like cells (Day 34) generated using differentiation protocol 2 showing staining for INS and NKX6-1 (G) and INS and GCG (H). Representative plots from different differentiations. I. Results from static glucose stimulated insulin secretion assay from American Indian hiPSC generated islets using differentiation protocol 2. Insulin secretion is shown as stimulation index; fold increase in insulin secretion when islets are stimulated with high glucose (20mM) or KCl (30mM) relative to insulin secretion when stimulated with low glucose (2mM). Results are from islets generated from two independent differentiations.

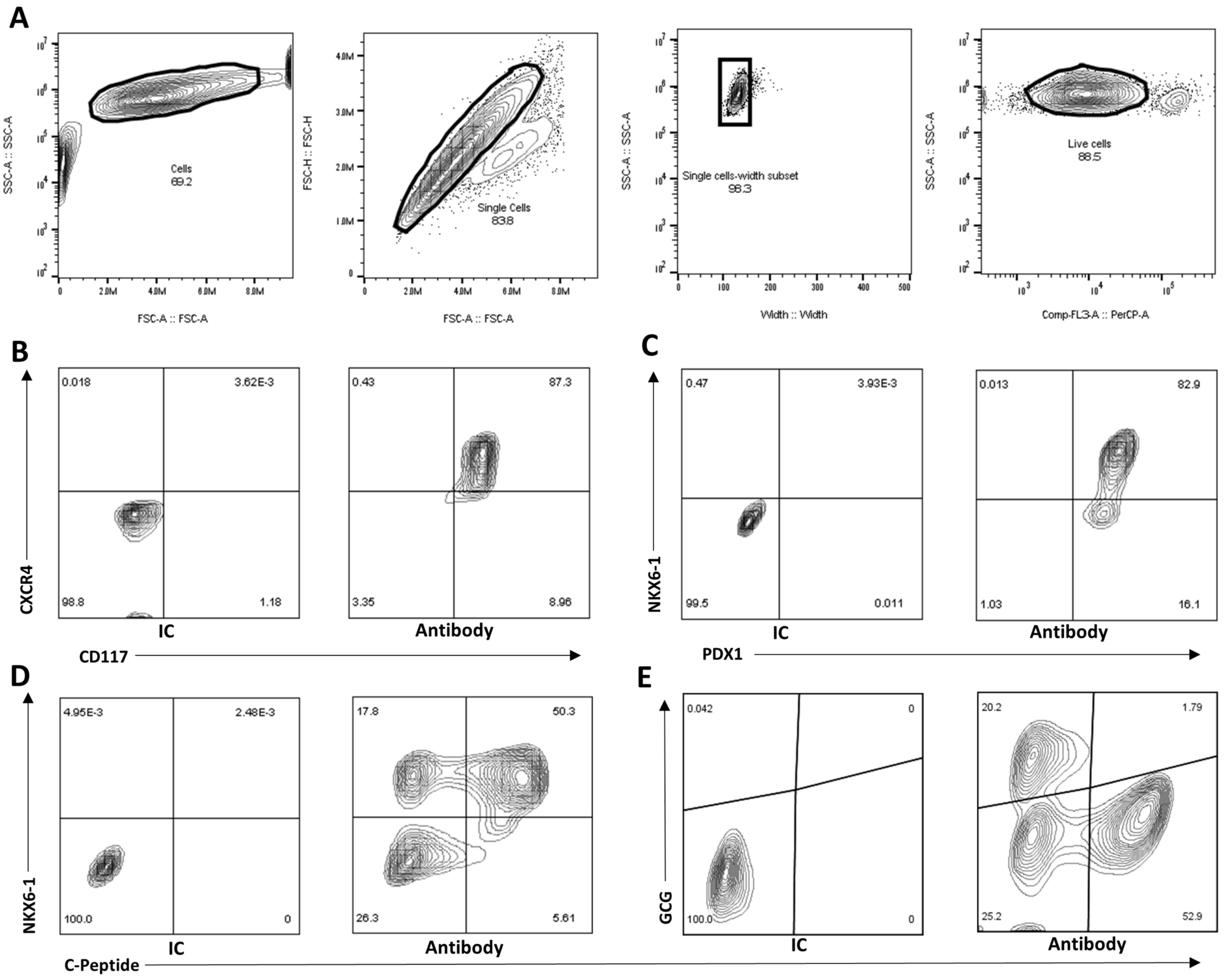

Figure S4. Gating strategy used for flow cytometry analysis and representative flow cytometry data from different stages of differentiation. **A**. Gating strategy used for identification of live single cells after the intracellular staining protocol. For surface marker staining, cells were identified using the SSC-A/FSC-A and FSC-A/FSC-H parameters. **B-E** Representative flow cytometry data showing staining of CXCR4/CD117 (B, definitive endoderm markers-day 2), PDX1-NKX6-1 (C, pancreatic progenitor markers-day 10), Insulin (C-peptide)/NKX6-1 (D, figure also shown in main text) and Insulin/Glucagon (E, pancreatic islet markers - day27-34) during different stages of differentiation along with the respective isotype controls (IC) used to determine gating. Data shown are from different differentiations.

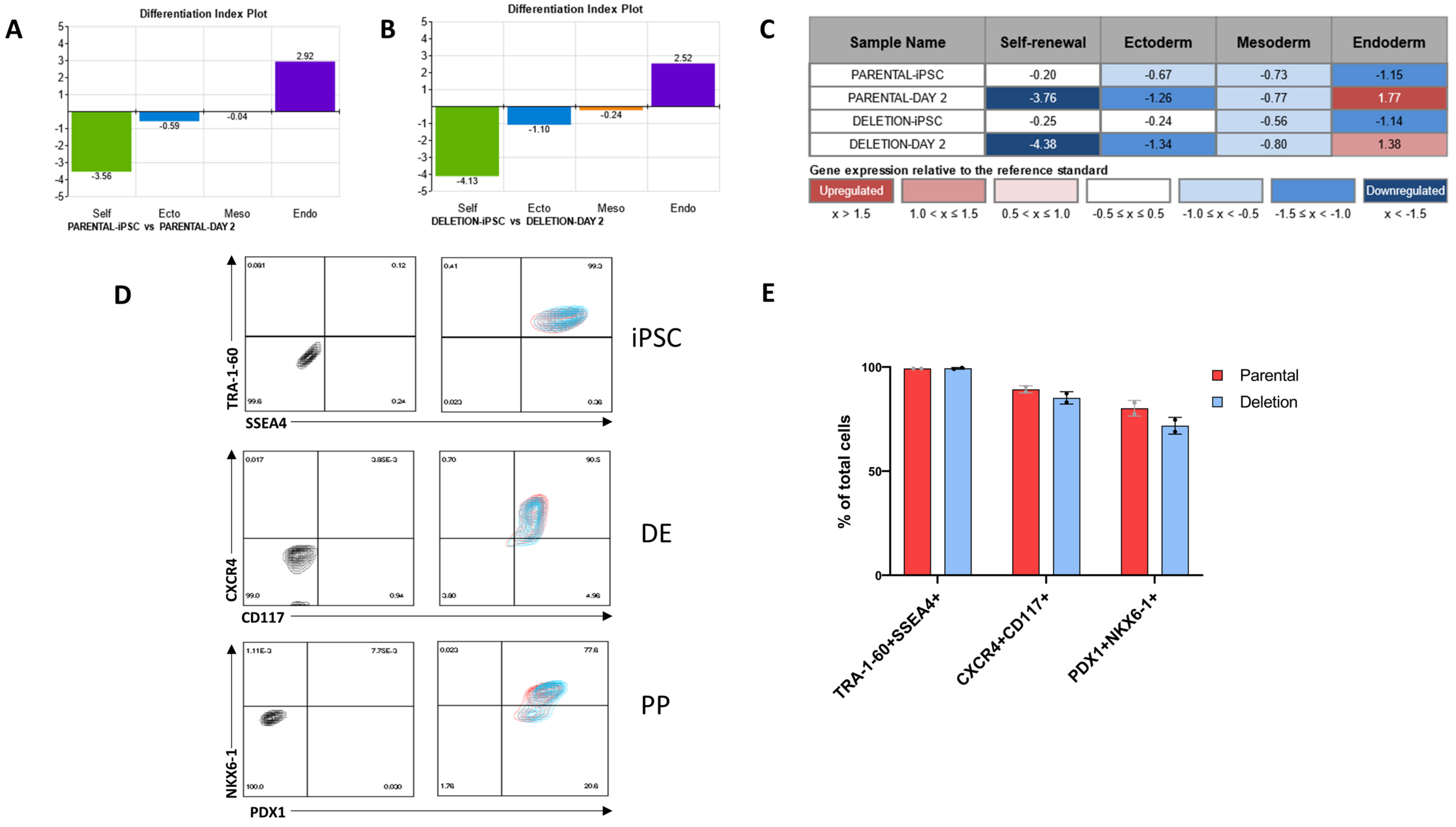

Figure S5. Pluripotency and differentiation potential of the parental and the deletion (sg3-C10) hiPSCs. **A-B**. Differentiation index plot generated from hPSC scorecard results after two days of directed differentiation of the parental hiPSCs (A) and deletion hiPSCs (B) towards definitive endoderm. Figure shows the downregulation (of algorithmic score) of self-renewal genes (pluripotency markers) and upregulation of definitive endoderm markers in the day 2 (definitive endoderm) cells compared to hiPSCs. **C**. Algorithmic scores from hPSC scorecard assay after two days of directed differentiation towards definitive endoderm. Reference standard – established human embryonic stem cell lines. **D-E**. Flow cytometry analysis to assess expression of pluripotency markers (TRA-1-60 and SSEA4), definitive endoderm markers (day 2 of differentiation, CXCR4 and CD117) and pancreatic progenitor markers (day 10 of differentiation, PDX1 and NKX6-1) in the parental cell line and the deletion cell line. IC – isotype control. Light red – parental cell line, light blue – deletion cell line.

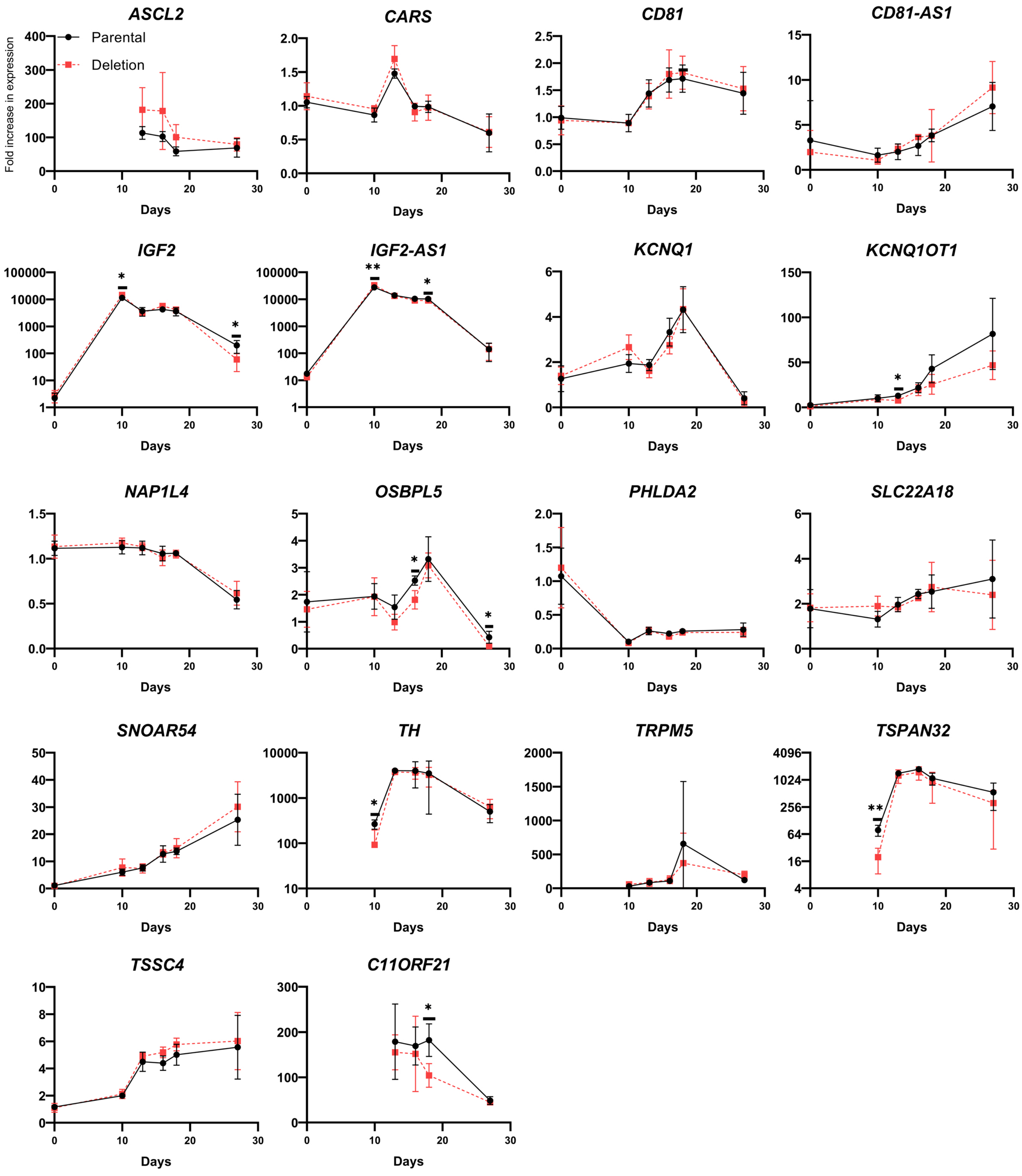

Figure S6. Stage-specific expression of genes at the *KCNQ1* locus during different stages of differentiation from the parental hiPSC and deletion hiPSC. Figure shows the fold increase in expression of genes at the *KCNQ1* locus (identified to be expressed during any stage of islet-development from hiPSCs, Figure 1B) during differentiation of the hiPSC with the deletion and the parental hiPSC to pancreatic islet-like cells. Results are from four independent differentiations and the error bars show standard deviation. Results were compared using t-test. Expression was calculated relative to expression in parental hiPSC from experiment 1. *P<0.05, **P<0.005.

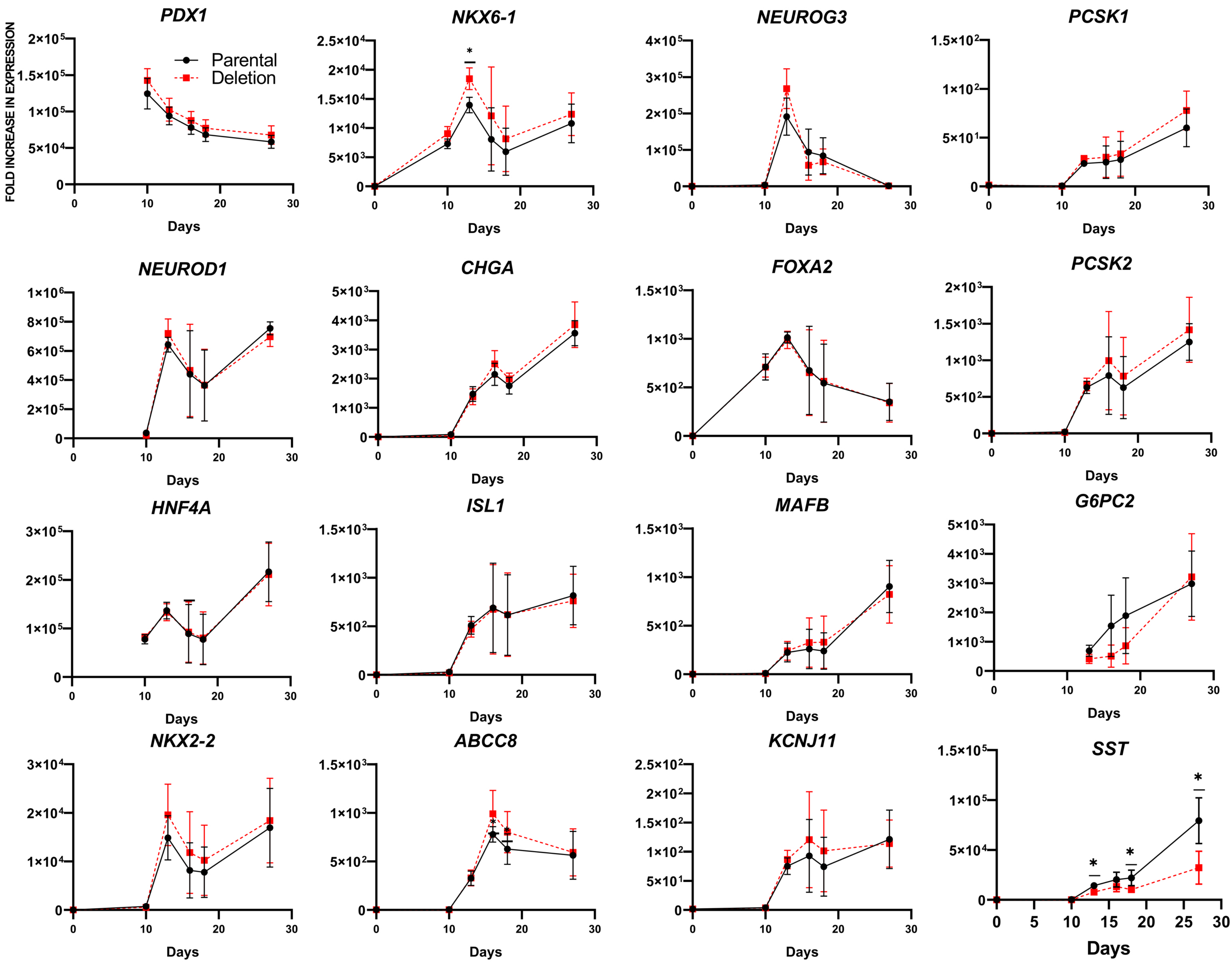

Figure S7. Stage specific expression of pancreatic islet differentiation markers during different stages of differentiation from the parental hiPSC and deletion hiPSC. Figure shows the fold increase in expression of pancreatic islet differentiation markers during differentiation of the deletion iPS cell line and the parental iPS cell line towards pancreatic islet-like cells. Results are from four independent differentiations and the error bars show SD. Expression was calculated relative to expression in parental hiPSC from experiment 1 and results were compared using t-test. *P<0.05. P values are shown for differences in fold change in expression only after day 10.
